# Efficacy and safety of probiotics in the treatment of iron deficiency anemia in Chinese children: a systematic review and meta-analysis

**DOI:** 10.3389/fnut.2026.1876092

**Published:** 2026-07-30

**Authors:** Gang Hu, Yunfeng Yu, Xinyu Yang, Di Zhang, Mengqing Wang, Fan Li

**Affiliations:** 1 Department of Pediatrics, The First Hospital of Hunan University of Chinese Medicine, Changsha, Hunan, China; 2 School of Traditional Chinese Medicine, Hunan University of Chinese Medicine, Changsha, Hunan, China

**Keywords:** iron deficiency anemia, meta-analysis, pediatric populations, probiotics, systematic review

## Abstract

**Objective:**

This meta-analysis, combined with trial sequential analysis (TSA), aimed to evaluate the effects of probiotics on hematologic parameters and iron-regulation biomarkers in Chinese children with iron deficiency anemia (IDA).

**Methods:**

Ten electronic databases were systematically searched for eligible studies published up to April 30, 2026. Meta-analyses were conducted using RevMan (version 5.3), and TSA was performed with TSA software (version 0.9.5.10 beta). Dichotomous and continuous outcomes were pooled as risk ratios (RRs) and mean differences (MDs), respectively. Publication bias was assessed using Egger’s and Harbord’s tests.

**Results:**

Twelve randomized controlled trials involving 1,342 children with IDA were included. In terms of efficacy, probiotics significantly increased hemoglobin (Hb) (MD 8.64 g/L, 95% CI 6.77–10.52), red blood cell (MD 0.47 × 10^12^/L, 95% CI 0.19–0.76), mean corpuscular hemoglobin concentration (MD 15.66 g/L, 95% CI 12.55–18.77), mean corpuscular volume (MCV) (MD 6.68 fL, 95% CI 4.69–8.67), mean corpuscular hemoglobin (MD 2.40 pg, 95% CI 1.57–3.22), and hematocrit (MD 3.36%, 95% CI 2.31–4.41), serum iron (MD 1.70 μmol/L, 95% CI 0.89–2.51), and serum ferritin (MD 5.62 μg/L, 95% CI 4.24–7.00), while significantly reducing transferrin levels (MD – 0.24 g/L, 95% CI –0.34 to –0.15). Regarding safety, probiotics significantly reduced the incidence of total adverse events (RR 0.52, 95% CI 0.28–0.97), with no significant effects observed on anorexia, nausea and vomiting, metallic taste, abdominal pain, diarrhea, or constipation. TSA demonstrated that all positive outcomes, except total adverse events, reached the required information size and were conclusive. No significant publication bias was detected for most outcomes. Although publication bias was observed for MCV, trim-and-fill analysis confirmed the robustness of the pooled effect estimate. The evidence certainty of all outcomes was low or very low.

**Conclusion:**

Probiotics may improve anemia-related parameters and iron metabolism in Chinese children with IDA. However, the pooled increase in Hb remains below the minimal clinically important difference, and evidence on total adverse events remains inconclusive. Therefore, probiotics should be considered as an adjunct to oral iron supplementation rather than a standalone intervention. These findings should be interpreted with caution due to potential biases and the inclusion of only Chinese children.

## 1 Introduction

Iron deficiency anemia (IDA) is a microcytic hypochromic anemia caused by insufficient body iron stores, leading to reduced hemoglobin (Hb) synthesis (1, 2). It is the most common micronutrient deficiency worldwide (1) and is particularly prevalent among preschool-aged children and women (3, 4). The global prevalence of IDA among children under 5 years of age has been reported to be 16.42% (5), while the prevalence among children under 6 years of age in China is approximately 20.61%, representing a substantial public health burden (6). Children with IDA may present with pallor, poor appetite, fatigue, lethargy, and dizziness, and severe cases may develop tachycardia and dyspnea (7, 8). Furthermore, prolonged or severe IDA has been associated with potentially irreversible impairments in neurodevelopment and cognitive function (9, 10). Currently, oral iron supplementation remains the first-line therapy for IDA (11). However, its effectiveness may be limited by inadequate intestinal absorption and gastrointestinal adverse events, which can reduce treatment adherence and compromise therapeutic outcomes (12–14). Therefore, there is an urgent need to explore safe and effective adjunctive therapies to further improve clinical outcomes in pediatric patients with IDA while minimizing iron-related gastrointestinal side effects.

The gut microbiota refers to the complex community of microorganisms colonizing the host gastrointestinal tract, including bacteria, fungi, and viruses (15). Accumulating evidence suggests that the gut microbiota can enhance host iron acquisition by reducing the concentration of iron-binding compounds in the intestinal lumen and promoting the conversion of ferric iron to ferrous iron (16, 17). Given its regulatory effects on iron metabolism, probiotics have been proposed as a potential strategy to improve iron absorption and alleviate iron deficiency (18). Clinical evidence suggests that probiotic supplementation can enhance non-heme dietary iron absorption, improve hematological outcomes, and reduce iron-related gastrointestinal adverse events in patients with IDA (19–21). Collectively, these findings provide preliminary support for the use of probiotics in enhancing iron absorption and improving anemia outcomes.

However, previous meta-analyses have had several limitations. The meta-analysis by Vonderheid et al. (19) included both adult and children, which may have increased clinical heterogeneity and limited the applicability of the findings. In addition, the analysis by Tremblay et al. (21) included only three clinical trials, and the limited evidence base may have affected the reliability of the conclusions. Given the high prevalence of IDA among Chinese children and potential differences in dietary patterns, nutritional status, and healthcare practices across populations, evidence from Chinese children may provide more clinically relevant insights for local practice. Therefore, this study focused on Chinese children with IDA and evaluated the efficacy and safety of probiotics combined with iron supplementation compared with iron supplementation alone. Trial sequential analysis (TSA) was further performed to assess the robustness of the pooled evidence and strengthen the interpretation of the findings. These methodological approaches were intended to provide more precise, objective, and comprehensive evidence to inform the clinical application of probiotics in the management of IDA in Chinese children.

## 2 Methods

This systematic review and meta-analysis was designed and reported in accordance with the Preferred Reporting Items for Systematic Reviews and Meta-Analyses (PRISMA) statement (22). The study protocol was prospectively registered in the PROSPERO database (registration number: CRD420251186010). The initial search was conducted on October 21, 2025, and the updated search was conducted on April 30, 2026.

### 2.1 Inclusion and exclusion criteria

Inclusion criteria: (i) Participants: Chinese children aged 0 to 18 years diagnosed with IDA according to international or national diagnostic standards, with no restrictions on gender, ethnicity, or disease duration. (ii) Interventions: Probiotic supplementation administered in combination with iron supplementation. The iron supplementation will adhere to the guidelines set forth by the “Expert Consensus on the Prevention and Treatment of Iron Deficiency and Iron Deficiency Anemia in Children” published by the Chinese Association of Maternal and Child Health Care (23). This includes ferrous sulfate, ferrous lactate, ferrous gluconate, ferrous fumarate, heme iron preparations, dextran iron dispersible tablets, amino acid chelated iron, and protein succinate iron. (iii) Comparisons: Iron supplementation alone, following the above guidelines. (iv) Outcomes: Anemia-related parameters included Hb, red blood cell (RBC), mean corpuscular hemoglobin concentration (MCHC), mean corpuscular volume (MCV), mean corpuscular hemoglobin (MCH), and hematocrit (HCT); Iron metabolism parameters included serum iron (SI), serum ferritin (SF), and transferrin (TF). The safety outcome was total adverse events, anorexia, nausea and vomiting, metallic taste, abdominal pain, diarrhea, and constipation. (v) Study design: Randomized controlled trials (RCTs) only.

Exclusion criteria: duplicate or retracted publications, studies with unavailable full texts or insufficient data, and studies containing obvious statistical inconsistencies were excluded.

### 2.2 Literature search strategy

A systematic and comprehensive literature search was conducted across 10 electronic databases, including China National Knowledge Infrastructure (CNKI), Wanfang Data, China Science and Technology Journal Database (CSTJ), PubMed, Embase, Web of Science, the Cochrane Library, ClinicalTrials.gov, Chinese Clinical Trial Registry (ChiCTR) and World Health Organization International Clinical Trials Registry Platform (WHO ICTRP), to ensure the comprehensiveness of the search strategy. In particular, the ChiCTR and WHO ICTRP facilitate the identification of unpublished clinical trials, thereby reducing the risk of publication bias. The search strategy was constructed by combining controlled subject vocabulary and free-text terms related to IDA and probiotics, including various probiotic genera and species. All the above terms were sourced from the Medical Subject Headings (MeSH) database and the Cochrane Library, and the full detailed search strategy is shown in Table 1. All databases were searched from their inception to April 30, 2026, without restrictions on language or publication status. The search strategy, filter, and restrictions for each database are presented in Supplementary Table S1. In addition, the reference lists of relevant reviews and all included studies were manually examined to identify additional potentially eligible trials.

**Table 1:** Search strategy.

Number	English terms	Chinese terms
#1	Iron Deficiency Anemia	缺铁性贫血
#2	Iron Deficiency Anemias OR Iron-Deficiency Anemia OR Iron Deficiency Anemia	铁缺乏性贫血 OR 营养性贫血
#3	#1 OR #2	#1 OR #2
#4	Probiotics	益生菌
#5	Probiotic OR Bifidobacterium OR Bifidobacteria OR Bacillus bifida OR yeast OR *Saccharomyces cerevisiae* OR Saccharomyces italicus OR Saccharomyces oviformis OR S cerevisiae OR *S. cerevisiae* OR Saccharomyces uvarum var. melibiosus OR *Candida robusta* OR Saccharomyces capensis OR *Lactobacillus acidophilus* OR *Lactobacillus amylovorus* OR Lactobacill OR Lactic acid bacteria OR *Clostridium butyricum* OR Bacillus OR Natto Bacteria OR Streptococcus thermophiles OR Enterococcus	微生态制剂 OR 双歧杆菌 OR 酵母菌 OR 嗜酸乳杆菌 OR 乳酸菌 OR 酪酸梭菌 OR 丁酸梭菌 OR 芽孢杆菌 OR 枯草杆菌 OR 链球菌
#6	#4 OR #5	#4 OR #5
#7	#3 AND #6	#3 AND #6

### 2.3 Study selection process

All records retrieved from the database searches were imported into a reference management tool (24). After automatic and manual removal of duplicate entries, two reviewers independently screened titles and abstracts to exclude clearly irrelevant studies. Full-text articles were then obtained for all potentially eligible records and assessed independently against the predefined eligibility criteria. Any disagreements during the selection process were resolved through discussion, and unresolved discrepancies were adjudicated by a third reviewer. The selection process was summarized using a PRISMA flow diagram.

### 2.4 Data extraction

Two reviewers independently extracted data using a predesigned and standardized extraction form (25). Extracted information included publication details, sample size, participant characteristics, intervention protocols, comparator treatments, and outcome data. When discrepancies arose, consensus was reached through discussion (26). If necessary, corresponding authors were contacted to obtain missing or unclear information.

### 2.5 Risk of bias assessment

The internal validity of each included RCT was independently evaluated by two reviewers using the Cochrane Risk of Bias tool (27). The following domains were assessed: random sequence generation, allocation concealment, blinding of participants and personnel, blinding of outcome assessment, completeness of outcome data, selective reporting, and other potential biases. Each domain was judged as having a low, high, or unclear risk of bias. Any disagreements were resolved through consensus or consultation with a third reviewer.

### 2.6 Data synthesis and statistical analysis

Quantitative synthesis was performed using Review Manager software (RevMan, version 5.3). For dichotomous outcomes, effect estimates were expressed as risk ratios (RRs) with corresponding 95% confidence intervals (CIs) (22). Continuous outcomes were summarized using mean differences (MDs) with 95% CIs. Statistical heterogeneity among studies was evaluated using Cochran’s *Q* test and quantified with the *I*^2^ statistic. An *I*^2^ value below 50% combined with a *p*-value of at least 0.10 was interpreted as low heterogeneity (28). Given the variability in probiotic strains, probiotic dosages, and treatment durations across studies, a random-effects model was applied for all meta-analyses.

To explore potential sources of heterogeneity, subgroup analyses were conducted for the primary outcome according to clinically relevant factors, including participant age, disease duration, baseline Hb levels, treatment duration, probiotic strains, and probiotic dosage (29). Sensitivity analyses were carried out by sequentially excluding individual studies, particularly those with small sample sizes, high risk of bias, or outlying effect estimates, to assess the stability of the pooled results (30).

The TSA was undertaken using TSA software (version 0.9.5.10 beta) to control for the risk of random errors due to repetitive testing and sparse data (31). The required information size (RIS) was calculated based on a two-sided alpha of 5%, a statistical power of 80%, and an anticipated intervention effect. Sequential monitoring boundaries were applied to determine whether the accumulated evidence was sufficient to draw firm conclusions or whether further trials were necessary. A result was considered conclusive if the cumulative *Z*-curve crossed the TSA monitoring boundary.

### 2.7 Assessment of publication bias

To quantitatively assess publication bias, both Egger’s test and Harbord’s test were applied according to the type of outcome measure (32, 33). For meta-analyses involving continuous outcomes, Egger’s test was used to evaluate funnel plot asymmetry. This method regresses the standard normal deviate of the effect size against its precision to test whether the intercept deviates significantly from zero. For dichotomous outcomes, Harbord’s test was employed. This test is a modification of Egger’s regression that adjusts for the variance structure of binary data, making it more robust for dichotomous outcomes. A *p*-value of less than 0.05 from Egger’s test and Harbord’s test was considered indicative of potential publication bias. When evidence of publication bias was detected, trim-and-fill method was applied to estimate the potential impact of missing studies on the pooled effect estimate.

### 2.8 Certainty of evidence

The overall certainty of evidence for each outcome was assessed using the Grading of Recommendations, Assessment, Development, and Evaluation (GRADE) approach (34). The assessment considered five domains: risk of bias, inconsistency, indirectness, imprecision, and publication bias. Based on these criteria, the certainty of evidence was classified as high, moderate, low, or very low, reflecting the level of confidence in the pooled effect estimates.

## 3 Results

### 3.1 Study selection

**Figure 1:**
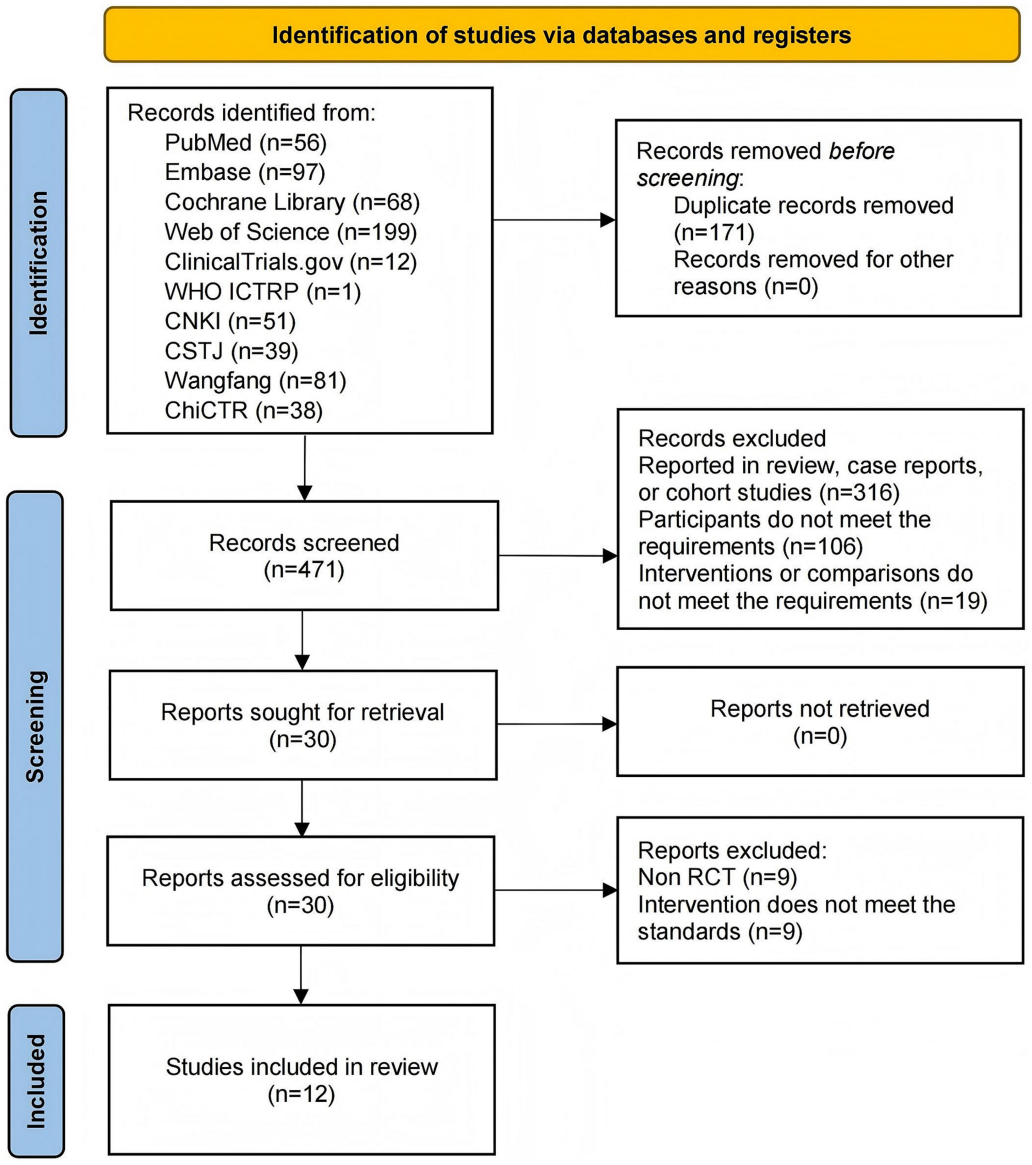
Literature screening process.

The systematic literature search identified a total of 642 records across all databases, including 51 from CNKI, 81 from Wanfang Data, 39 from CSTJ, 56 from PubMed, 97 from Embase, 199 from Web of Science, 68 from the Cochrane Library, 12 from ClinicalTrials.gov, 38 from ChiCTR, 1 from WHO ICTRP. After removal of 171 duplicate records, 471 articles remained for title and abstract screening. Of these, 402 records were excluded for the following reasons: 316 articles were reported in reviews, case reports, or cohort studies; 106 articles were excluded because the participants did not meet the requirements; and 19 articles were excluded because the interventions or comparisons did not meet the requirements. The full texts of the remaining studies were assessed for eligibility, resulting in the exclusion of 18 articles: nine were excluded because they were not randomized controlled trials, and nine did not meet the predefined intervention criteria. Ultimately, 12 studies (35–46) met the inclusion criteria and were included in the meta-analysis. The study selection process is illustrated in Figure 1.

### 3.2 Basic characteristics of included studies

A total of 12 RCTs comprising 1,342 pediatric participants were included in the meta-analysis. These studies were published between 2018 and 2024, and all were conducted in China. The proportion of male participants ranged from 45.83 to 63.16%. The mean age of participants varied from 4.50 months to 4.27 years. Two trials evaluated single-strain probiotic formulations, whereas the remaining 10 trials used multi-strain probiotic preparations. The intervention duration ranged from 4 to 8 weeks. Detailed characteristics of the included studies are presented in Table 2.

**Table 2:** Basic characteristics of included studies.

Author name	Sample size	Age (years)	Male (%)	Baseline Hb levels (g/L)	IDA duration (months)	Diagnostic criteria	Intervention	Treatment duration (weeks)
Zhuang (35)	75	2.13 ± 0.25	49.33	/	/	Prevention and Management of Iron Deficiency and Iron-Deficiency Anemia in Children	Eosinophil-Lactobacillus Compound tablets: Each 1 g contains *Lactobacillus acidophilus* 1 × 10^7^ CFU, 0.5 g Bid	4
	75	2.17 ± 0.29	46.67	/	/		Iron Dextran Dispersible Tablets, 25 mg Bid	
Han (36)	76	2.16 ± 0.68	63.16	90.41 ± 8.98	/	Prevention and Management of Iron Deficiency and Iron-Deficiency Anemia in Children	Eosinophil-Lactobacillus Compound tablets: Each 1 g contains *Lactobacillus acidophilus* 1 × 10^7^ CFU, 1 g Tid	8
	76	2.08 ± 0.71	57.89	91.12 ± 9.20	/		Iron Dextran Oral Solution, 5 mg/(kg·d) Tid	
Tang (37)	50	2.34 ± 1.13	56.00	90.13 ± 6.21	3.24 ± 0.78	Zhu Futang’s Practical Pediatrics, 8th Edition	Live Combined Bifidobacterium, Lactobacillus and Enterococcus Capsules, Oral: *Bifidobacterium longum* + *Lactobacillus acidophilus* + *Enterococcus faecium* ≥ 1.0 × 10^7^ CFU, 210 mg Bid	4
	50	2.35 ± 1.00	52.00	90.26 ± 6.56	3.21 ± 1.57		Iron Dextran Oral Solution, 5 mg/(kg·d) Tid	
Wang (38)	50	2.19 ± 1.03	54.00	90.14 ± 6.22	3.29 ± 1.91	Zhu Futang’s Practical Pediatrics, 8th Edition	Live Combined Bifidobacterium, Lactobacillus and Enterococcus Capsules, Oral: *Bifidobacterium longum* + *Lactobacillus acidophilus* + *Enterococcus faecium* ≥ 1.0 × 10^7^ CFU, 210 mg Bid	4
	50	2.23 ± 1.02	56.00	90.27 ± 6.57	3.33 ± 1.85		Iron Dextran Oral Solution, 5 mg/kg Tid	
Wang (39)	96	0.38 ± 0.40	45.83	76.52 ± 21.60	0.53 ± 0.17	Zhu Futang’s Practical Pediatrics, 8th Edition	Bifid Triple Viable Capsules Dissolving at Intestines: Each 1 g contains *Bifidobacterium longum* ≥ 1.0 × 10^6^ CFU + *Lactobacillus acidophilus* ≥ 1.0 × 10^6^ CFU + *Enterococcus faecium* ≥ 1.0 × 10^6^ CFU, 210–420 mg Qd	4
	96	0.42 ± 0.39	46.88	78.10 ± 22.7	0.53 ± 0.17		Iron Proteinsuccinylate Oral Solution, 2 mg/kg Qd	
Yang (40)	59	/	/	100.25 ± 5.26	/	Zhu Futang’s Practical Pediatrics, 7th Edition, Volumes I & II (Hardcover)	Live Combined Bifidobacterium, Lactobacillus and Enterococcus Capsules, Oral: *Bifidobacterium longum* + *Lactobacillus acidophilus* + *Enterococcus faecium* ≥ 1.0 × 10^7^ CFU, 210–240 mg Bid	8
	53	/	/	100.36 ± 5.17	/		Trivitamins Ferrous Chewable Tablets, <2Y 1 tablet; ≥2Y 2–4 tablet Bid/Tid	
Yang (41)	32	2.42 ± 0.39	59.38	90.89 ± 9.41	5.19 ± 1.45	Zhu Futang’s Practical Pediatrics, 8th Edition	Live Combined Bifidobacterium, Lactobacillus and Enterococcus Capsules, Oral: *Bifidobacterium longum* + *Lactobacillus acidophilus* + *Enterococcus faecium* ≥ 1.0 × 10^7^ CFU, 210 mg Bid	8
	32	2.35 ± 0.44	53.13	88.25 ± 8.56	5.25 ± 1.34		Multivitamin Iron Oral Solution, 6 M-2Y 2–4 mL Bid; 2-5Y 5–7 mL Bid	
Gao (42)	41	0.54 ± 0.08	56.10	90.15 ± 12.55	0.47 ± 0.06	Multidisciplinary Expert Consensus on the Diagnosis, Treatment, and Prevention of Iron Deficiency and Iron-Deficiency Anemia (2022 Edition)	Bifid triple viable capsules dissolving at intestines: each 1 g contains *Bifidobacterium longum* ≥ 1.0 × 10^6^ CFU + *Lactobacillus acidophilus* ≥ 1.0 × 10^6^ CFU + *Enterococcus faecium* ≥ 1.0 × 10^6^ CFU, 210 mg Bid	4
	41	0.54 ± 0.09	51.22	90.25 ± 12.62	0.46 ± 0.05		Iron Dextran Oral Solution, 3.5 mg/(kg·d)	
Li (43)	56	3.81 ± 1.42	53.57	90.36 ± 7.45	15.02 ± 5.76	Zhu Futang’s Practical Pediatrics, 8th Edition	Live Combined bifidobacterium, Lactobacillus and Enterococcus Capsules, Oral: *Bifidobacterium longum* + *Lactobacillus acidophilus* + *Enterococcus faecium* ≥ 1.0 × 10^7^ CFU, 210 mg Bid	4
	56	3.62 ± 1.57	51.79	90.82 ± 7.06	15.23 ± 6.08		Iron Dextran Dispersible Tablets, 25–50 mg Tid	
Lv (44)	47	2.69 ± 0.53	55.32	88.69 ± 8.69	5.01 ± 1.08	Zhu Futang’s Practical Pediatrics, 8th Edition	Live Combined Bifidobacterium, Lactobacillus and Enterococcus Capsules, Oral: *Bifidobacterium longum* + *Lactobacillus acidophilus* + *Enterococcus faecium* ≥ 1.0 × 10^7^ CFU, 210 mg Bid	8
	47	2.67 ± 0.52	53.19	88.34 ± 8.67	5.03 ± 1.02		Multivitamin Iron Oral Solution, <2Y 2–4 mL bid; 2-5Y 5-7 mL Bid	
Fu (45)	38	2.63 ± 0.58	52.63	91.43 ± 11.54	4.12 ± 0.72	Zhu Futang’s Practical Pediatrics, 8th Edition	Live Combined Bifidobacterium, Lactobacillus and Enterococcus Powder, Oral: Each 1 g contains *Bifidobacterium longum* + *Lactobacillus acidophilus* + *Enterococcus faecium* ≥ 1.0 × 10^7^ CFU, 2 g Tid	4
	38	2.73 ± 0.65	57.89	92.14 ± 12.36	4.35 ± 0.67		Iron Dextran Oral Solution, 2 mg/kg Tid	
Zhang (46)	54	4.27 ± 1.32	62.96	78.92 ± 10.83	12.62 ± 3.93	Zhu Futang’s Practical Pediatrics, 7th Edition	Bifid Triple Viable Capsules Dissolving at Intestines: Each 1 g contains *Bifidobacterium longum* ≥ 1.0 × 10^6^ CFU + *Lactobacillus acidophilus* ≥ 1.0 × 10^6^ CFU + *Enterococcus faecium* ≥ 1.0 × 10^6^ CFU, 210 mg Bid	8
	54	4.21 ± 1.54	61.11	78.67 ± 10.91	12.67 ± 3.99		Iron Proteinsuccinylate Oral Solution, 1.5 mL/kg Bid	

### 3.3 Risk of bias

**Figure 2:**
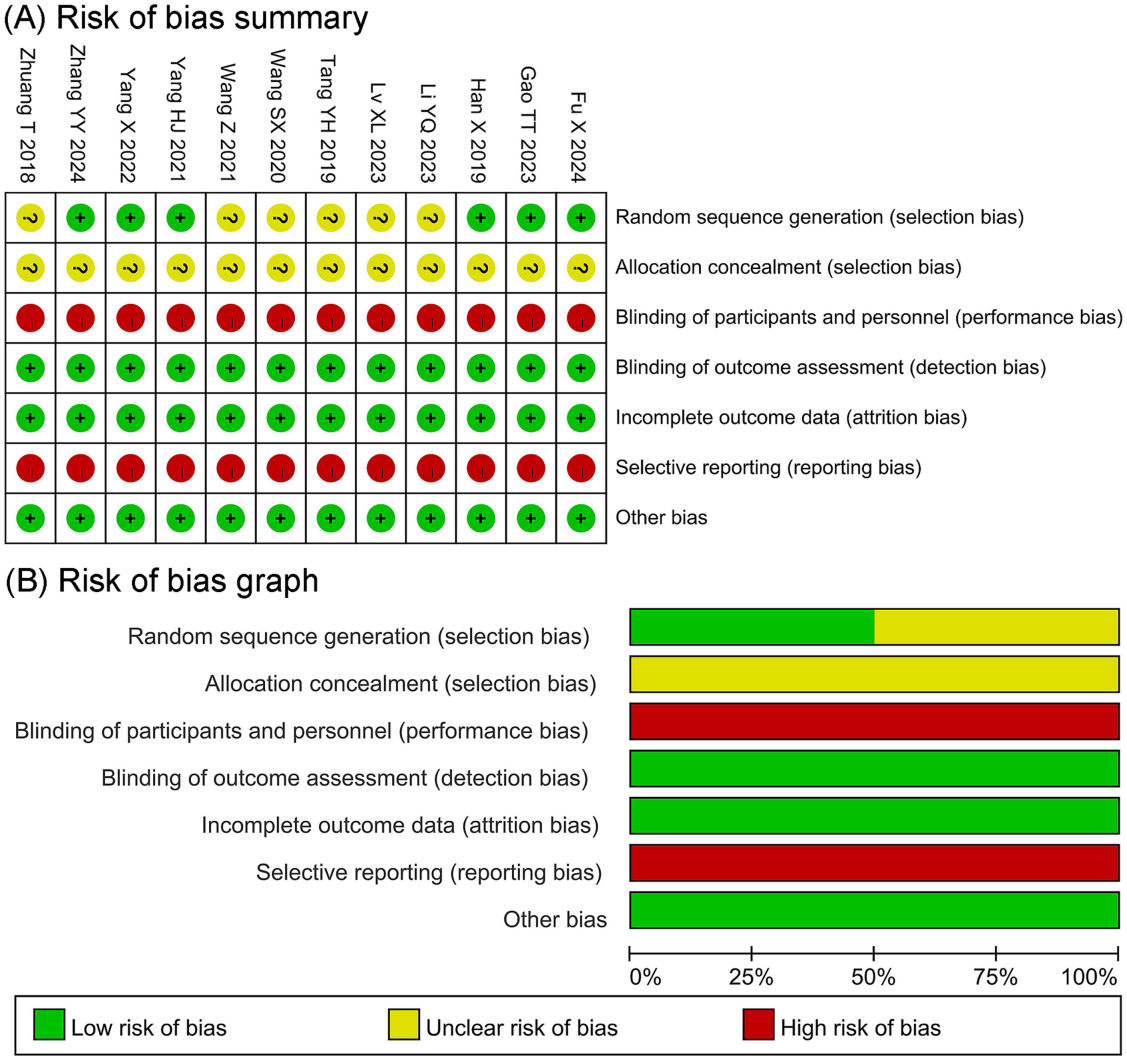
Risk assessment of bias. **(A)** Risk of bias summary; **(B)** risk of bias graph.

The risk of bias assessment is summarized in Figure 2. Among the 12 included trials, six did not clearly describe the method used for random sequence generation. Allocation concealment was not reported in any of the studies, and none of the trials implemented blinding procedures. However, as the primary outcomes were measured using objective laboratory indicators, the risk of detection bias was considered low. All studies reported attrition rates below 20%, indicating a low risk of attrition bias. None of the included trials provided a preregistered protocol or trial registration record, making it impossible to verify whether all prespecified outcomes were fully reported. Therefore, the risk of selective reporting bias was judged to be high. No other obvious sources of bias were identified.

### 3.4 Meta-analysis

#### 3.4.1 Anemia-related parameters

**Figure 3:**
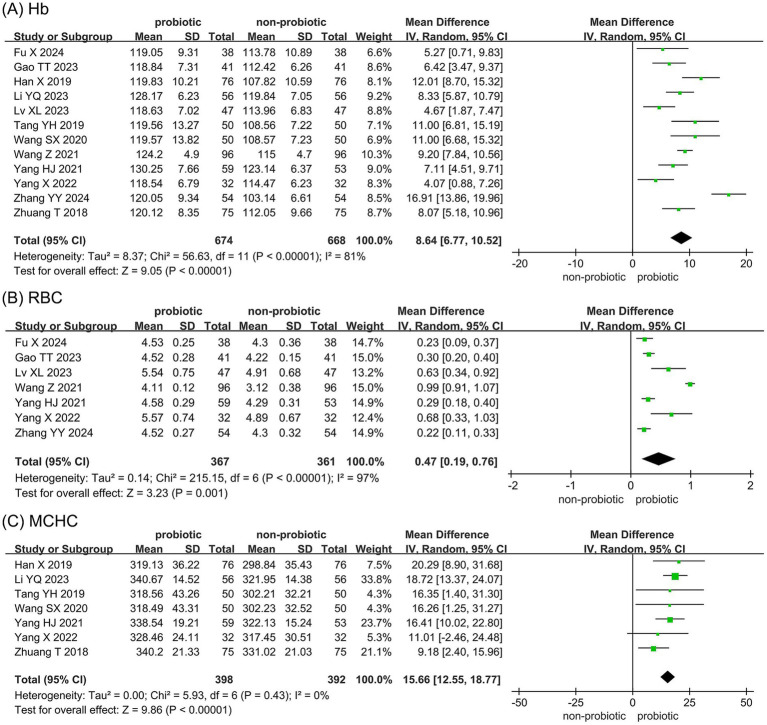
Forest plots of the meta-analysis on Hb, RBC, and MCHC: **(A)** Hb; **(B)** RBC; **(C)** MCHC. Hb, hemoglobin; RBC, red blood cell count; MCHC, mean corpuscular hemoglobin concentration.

*Hb*: The meta-analysis for Hb levels included 12 studies involving 1,342 participants. The Cochran’s *Q* test and the *I*^2^ statistic indicated high heterogeneity (*p* < 0.00001, *I*^2^ = 81%). The results demonstrated a significant increase in Hb levels in the probiotic group compared to the non-probiotic group (MD 8.64 g/L, 95% CI 6.77–10.52, *p* < 0.00001; Figure 3A). However, the observed improvement did not reach the minimal clinically important difference for Hb in children with IDA, defined as 10 g/L (1.0 g/dL).

*RBC*: The meta-analysis for RBC levels included seven studies involving 728 participants. The Cochran’s *Q* test and the *I*^2^ statistic indicated high heterogeneity (*p* < 0.00001, *I*^2^ = 97%). The results demonstrated that the probiotic group significantly increased RBC levels compared to the non-probiotic group (MD 0.47 × 10^12^/L, 95% CI 0.19–0.76, *p* = 0.001; Figure 3B).

*MCHC*: The meta-analysis for MCHC levels included seven studies involving 790 participants. The Cochran’s *Q* test and the *I*^2^ statistic indicated no significant heterogeneity (*p* = 0.43, *I*^2^ = 0%). The results revealed that the probiotic group significantly increased MCHC levels compared to the non-probiotic group (MD 15.66 g/L, 95% CI 12.55–18.77, *p* < 0.00001; Figure 3C).

**Figure 4:**
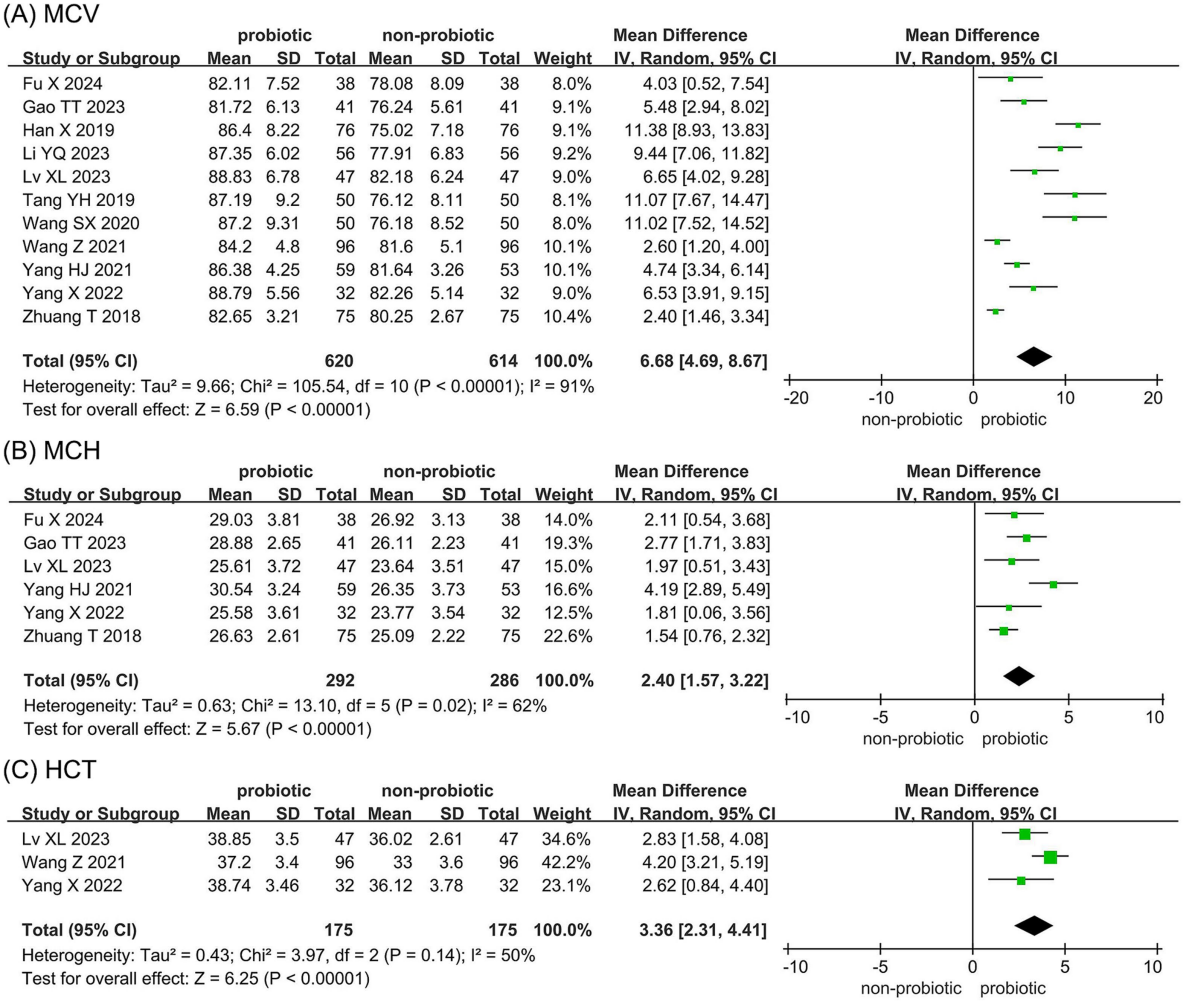
Forest plots of the meta-analysis on MCV, MCH, and HCT: **(A)** MCV; **(B)** MCH; **(C)** HCT. MCV, mean corpuscular volume; MCH, mean corpuscular hemoglobin; HCT, hematocrit.

*MCV*: The meta-analysis for MCV levels included 11 studies involving 1,234 participants. The Cochran’s *Q* test and the *I*^2^ statistic indicated high heterogeneity (*p* < 0.00001, *I*^2^ = 91%). The results revealed that the probiotic group significantly increased MCV levels compared to the non-probiotic group (MD 6.68 fL, 95% CI 4.69–8.67, *p* < 0.00001; Figure 4A).

*MCH*: The meta-analysis for MCH levels included six studies involving 578 participants. The Cochran’s *Q* test and the *I*^2^ statistic indicated high heterogeneity (*p* = 0.02, *I*^2^ = 62%). The results showed a significant increase in MCH levels in the probiotic group compared to the non-probiotic group (MD 2.40 pg, 95% CI 1.57–3.22, *p* < 0.00001; Figure 4B).

*HCT*: The meta-analysis for HCT levels included three studies involving 350 participants. The Cochran’s *Q* test and the *I*^2^ statistic indicated no significant heterogeneity (*p* = 0.14, *I*^2^ = 50%). The results showed a significant increase in HCT levels in the probiotic group compared to the non-probiotic group (MD 3.36%, 95% CI 2.31–4.41, *p* < 0.00001; Figure 4C).

#### 3.4.2 Iron metabolism parameters

**Figure 5:**
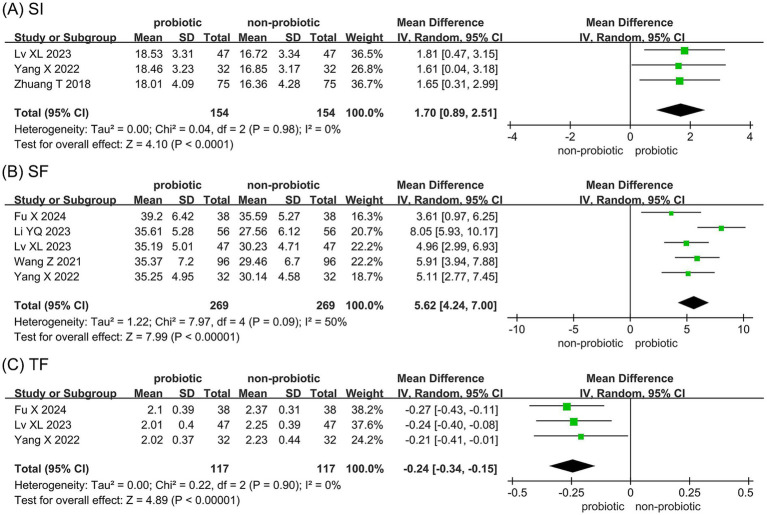
Forest plots of the meta-analysis on iron metabolism parameters: **(A)** SI; **(B)** SF; **(C)** TF. SI, serum iron; SF, serum ferritin; TF, transferrin.

*SI*: The meta-analysis for SI levels included three studies involving 308 participants. The Cochran’s *Q* test and the *I*^2^ statistic indicated no significant heterogeneity (*p* = 0.98, *I*^2^ = 0%). The results demonstrated that the probiotic group significantly increased SI levels compared to the non-probiotic group (MD 1.70 μmol/L, 95% CI 0.89–2.51, *p* < 0.0001; Figure 5A).

*SF*: The meta-analysis for SF levels included five studies involving 538 participants. The Cochran’s *Q* test and the *I*^2^ statistic indicated high heterogeneity (*p* = 0.09, *I*^2^ = 50%). The results demonstrated that the probiotic group significantly increased SF levels compared to the non-probiotic group (MD 5.62 μg/L, 95% CI 4.24–7.00, *p* < 0.00001; Figure 5B).

*TF*: The meta-analysis for TF levels included three studies involving 234 participants. The Cochran’s *Q* test and the *I*^2^ statistic indicated no significant heterogeneity (*p* = 0.90, *I*^2^ = 0%). The results suggested a significant reduction in TF levels in the probiotic group compared to the non-probiotic group (MD –0.24 g/L, 95% CI –0.34 to –0.15, *p* < 0.00001; Figure 5C).

#### 3.4.3 Safety outcomes

**Figure 6:**
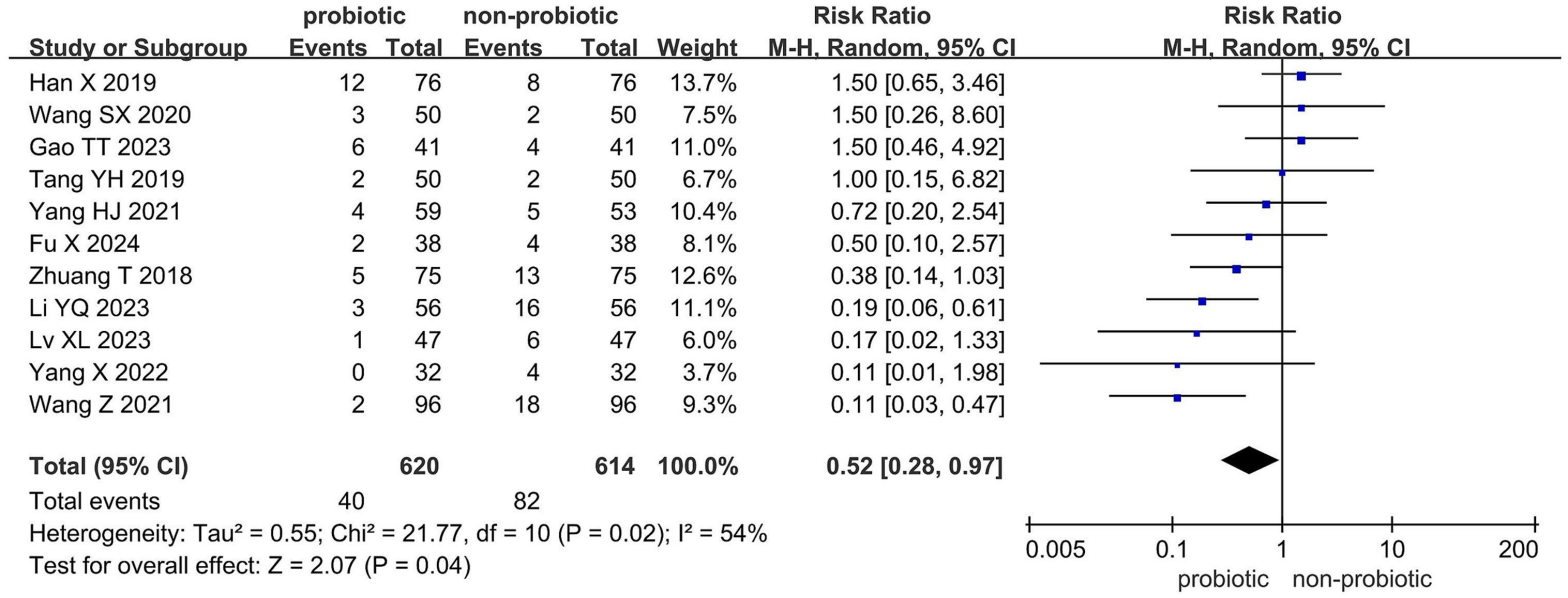
Forest plots of the meta-analysis on total adverse events.

*Total adverse events*: The meta-analysis for total adverse events included 11 studies with 1,234 participants. The total adverse events in the probiotic group was 6.45%, compared to 13.36% in the non-probiotic group. The Cochran’s *Q* test and the *I*^2^ statistic indicated high heterogeneity (*p* = 0.02, *I*^2^ = 54%). The results showed a significant decrease in total adverse events in the probiotic group compared to the non-probiotic group (RR 0.52, 95% CI 0.28–0.97, *p* = 0.04; Figure 6).

**Figure 7:**
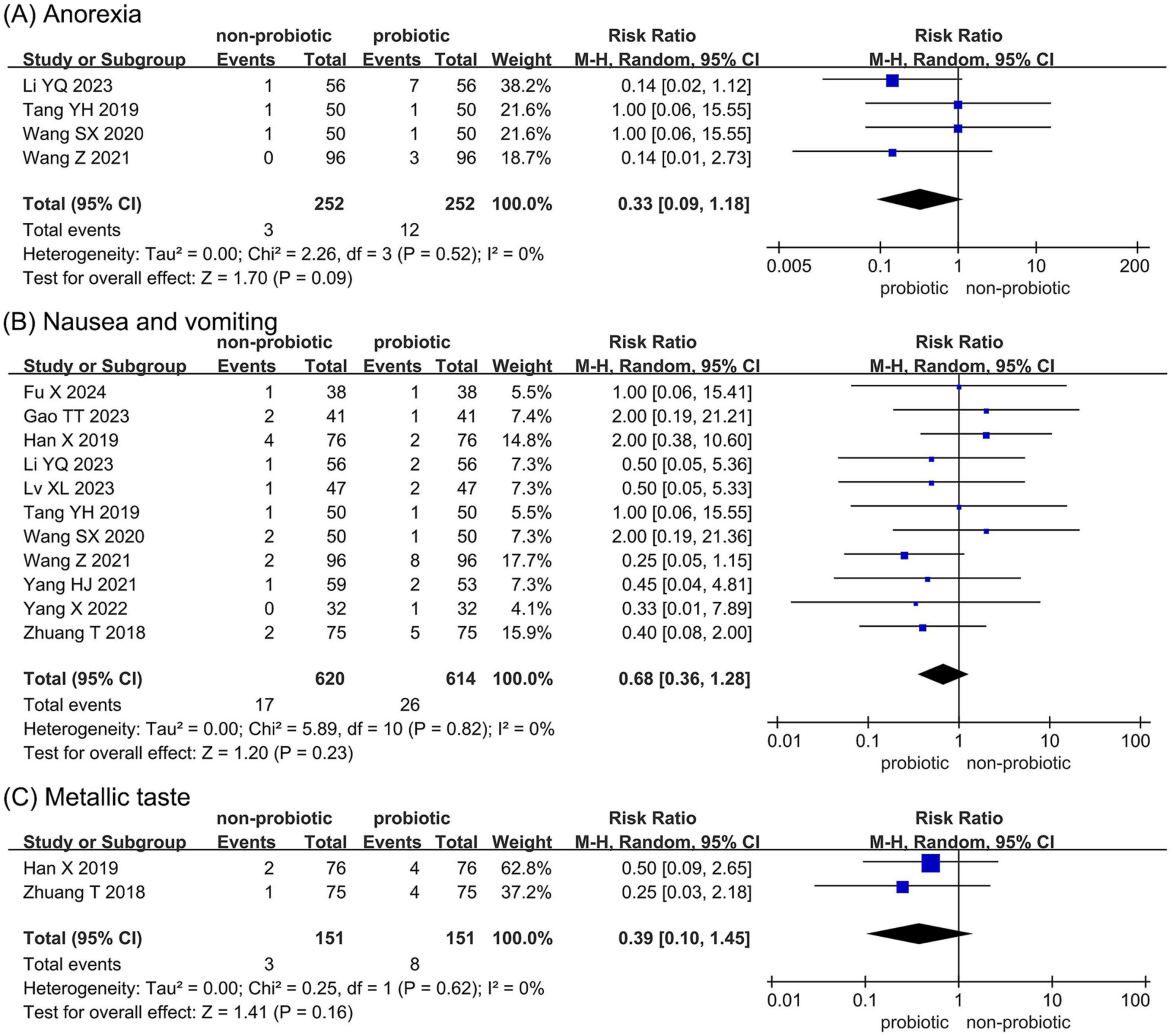
Forest plots of the meta-analysis on anorexia, nausea and vomiting, and metallic taste. **(A)** Anorexia; **(B)** nausea and vomiting; **(C)** metallic taste.

*Anorexia*: The meta-analysis for anorexia included four studies with 504 participants. The anorexia in the probiotic group was 1.19%, compared to 4.76% in the non-probiotic group. The Cochran’s *Q* test and the *I*^2^ statistic indicated no significant heterogeneity (*p* = 0.52, *I*^2^ = 0%). The results showed a significant decrease in anorexia in+ the probiotic group compared to the non-probiotic group (RR 0.33, 95% CI 0.09–1.18, *p* = 0.09; Figure 7A).

*Nausea and vomiting*: The meta-analysis for nausea and vomiting included 11 studies with 1,234 participants. The nausea and vomiting in the probiotic group was 2.74%, compared to 4.23% in the non-probiotic group. The Cochran’s *Q* test and the *I*^2^ statistic indicated no significant heterogeneity (*p* = 0.82, *I*^2^ = 0%). The results showed no statistically significant difference in nausea and vomiting between the probiotic and non-probiotic group (RR 0.68, 95% CI 0.36–1.28, *p* = 0.23; Figure 7B).

*Metallic taste*: The meta-analysis for metallic taste included two studies with 302 participants. The metallic taste in the probiotic group was 1.99%, compared to 5.29% in the non-probiotic group. The Cochran’s *Q* test and the *I*^2^ statistic indicated no significant heterogeneity (*p* = 0.62, *I*^2^ = 0%). The results showed no statistically significant difference in metallic taste between the probiotic and non-probiotic group (RR 0.39, 95% CI 0.10–1.45, *p* = 0.16; Figure 7C).

**Figure 8:**
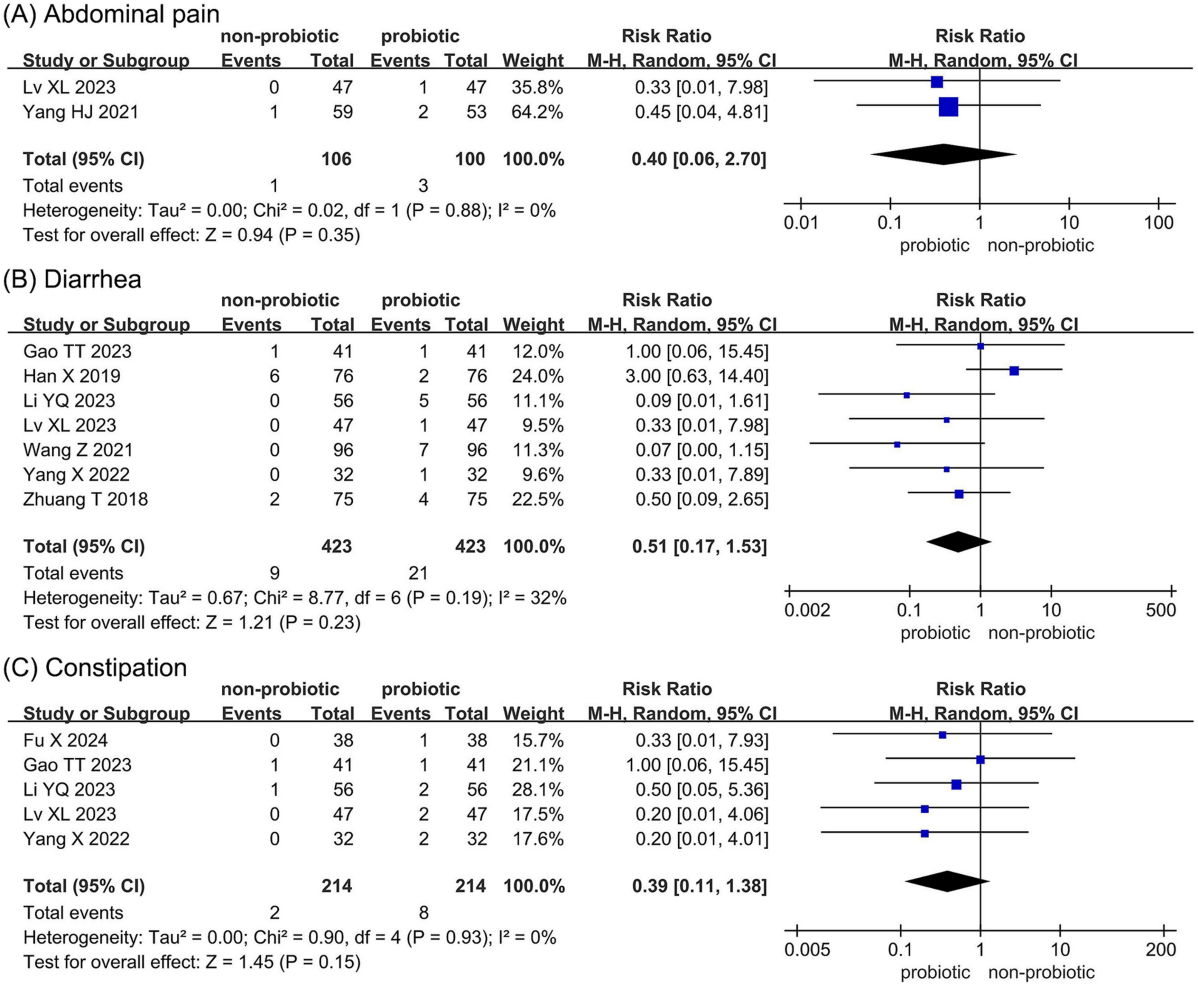
Forest plots of the meta-analysis on abdominal pain, diarrhea, and constipation. **(A)** Abdominal pain; **(B)** diarrhea; **(C)** constipation.

*Abdominal pain*: The meta-analysis for abdominal pain included two studies with 206 participants. The abdominal pain in the probiotic group was 0.94%, compared to 3.00% in the non-probiotic group. The Cochran’s *Q* test and the *I*^2^ statistic indicated no significant heterogeneity (*p* = 0.88, *I*^2^ = 0%). The results showed no statistically significant difference in abdominal pain between the probiotic and non-probiotic group (RR 0.40, 95% CI 0.06–2.70, *p* = 0.35; Figure 8A).

*Diarrhea*: The meta-analysis for diarrhea included seven studies with 846 participants. The diarrhea in the probiotic group was 2.13%, compared to 4.96% in the non-probiotic group. The Cochran’s *Q* test and the *I*^2^ statistic indicated no significant heterogeneity (*p* = 0.19, *I*^2^ = 32%). The results showed no statistically significant difference in diarrhea between the probiotic and non-probiotic group (RR 0.51, 95% CI 0.17–1.53, *p* = 0.23; Figure 8B).

*Constipation*: The meta-analysis for constipation included five studies with 428 participants. The constipation in the probiotic group was 0.93%, compared to 3.74% in the non-probiotic group. The Cochran’s *Q* test and the *I*^2^ statistic indicated no significant heterogeneity (*p* = 0.93, *I*^2^ = 0%). The results showed no statistically significant difference in constipation between the probiotic and non-probiotic group (RR 0.39, 95% CI 0.11–1.38, *p* = 0.15; Figure 8C).

### 3.5 Subgroup analysis

Substantial heterogeneity (*I*^2^ ≥ 50%) was observed in several outcomes, including Hb, RBC, MCV, MCH, SF, and total adverse events. Accordingly, subgroup analysis was conducted to investigate the effects of clinical factors such as average age, disease duration, baseline Hb levels, treatment duration, probiotic strains, probiotic dosage, and type of iron supplementation contributed to the heterogeneity, as detailed in Table 3.

**Table 3:** Subgroup analyses of Hb, RBC, MCV, MCH, SF, and total adverse events.

Primary outcome	Subject	Subgroup	Number of studies	*I*^2^/%	MD/RR (95% CI)	*p-*value
Hb	Average age	<2.5 years	7	65	8.66 (6.79–10.53)	<0.00001
		≥2.5 years	4	92	8.86 (3.39–14.33)	0.002
	Disease duration	<5 months	5	44	8.60 (6.72–10.48)	<0.00001
		≥5 months	4	93	8.49 (3.03–13.95)	0.002
	Baseline Hb levels	<90 g/L	3	94	10.20 (4.57–15.83)	0.0004
		≥90 g/L	7	50	8.61 (6.84–10.39)	<0.00001
	Treatment duration	4 weeks	7	20	8.55 (7.37–9.72)	<0.00001
		8 weeks	5	92	8.93 (4.28–13.59)	0.0002
	Probiotic strains	Single-strain	2	68	9.95 (6.10–13.81)	<0.00001
		Multi-strain	10	83	8.38 (6.22–10.54)	<0.00001
	Probiotic dosage	<1 × 10^7^ CFU/day	3	92	10.78 (5.70–15.85)	<0.0001
		≥1 × 10^7^ CFU/day	9	64	7.83 (6.03–9.62)	<0.00001
	Type of iron supplementation	Iron dextran	7	45	8.79 (7.07–10.51)	<0.00001
		Iron protein succinylate	2	95	12.93 (5.38–20.48)	0.0008
		Compound iron preparations	3	22	5.43 (3.57–7.30)	<0.00001
RBC	Average age	<2.5 years	3	98	0.66 (0.12–1.19)	0.02
		≥2.5 years	3	71	0.31 (0.13–0.49)	0.0005
	Disease duration	<5 months	3	99	0.51 (–0.01–1.03)	0.05
		≥5 months	3	83	0.48 (0.14–0.82)	0.005
	Baseline Hb levels	<90 g/L	3	98	0.61 (0.04–1.19)	0.04
		≥90 g/L	3	0	0.28 (0.22–0.35)	<0.00001
	Treatment duration	4 weeks	3	99	0.51 (–0.01–1.03)	0.05
		8 weeks	4	74	0.39 (0.22–0.56)	<0.0001
	Probiotic dosage	<1 × 10^7^ CFU/day	3	99	0.50 (–0.01–1.02)	0.05
		≥1 × 10^7^ CFU/day	4	71	0.40 (0.22–0.58)	<0.0001
	Type of iron supplementation	Iron dextran	2	0	0.28 (0.20–0.36)	<0.00001
		Iron protein succinylate	2	99	0.61 (–0.15–1.36)	0.12
		Compound iron preparations	3	75	0.50 (0.22–0.78)	0.0006
MCV	Average age	<2.5 years	7	93	6.99 (4.12–9.87)	<0.00001
		≥2.5 years	3	70	6.92 (3.96–9.88)	<0.00001
	Disease duration	<5 months	5	89	6.68 (3.15–10.21)	0.0002
		≥5 months	3	41	7.61 (5.69–9.53)	<0.00001
	Baseline Hb levels	<90 g/L	2	86	4.47 (0.51–8.42)	0.03
		≥90 g/L	7	85	8.09 (5.59–10.59)	<0.00001
	Treatment duration	4 weeks	7	91	6.36 (3.72–8.99)	<0.00001
		8 weeks	4	86	7.25 (4.31–10.19)	<0.00001
	Probiotic strains	Single-strain	2	98	6.82 (–1.98–15.61)	0.13
		Multi-strain	9	84	6.65 (4.71–8.59)	<0.00001
	probiotic dosage	<1 × 10^7^ CFU/day	2	74	3.84 (1.04–6.63)	0.007
		≥1 × 10^7^ CFU/day	9	92	7.35 (4.91–9.79)	<0.00001
	Type of iron supplementation	Iron dextran	7	94	7.76 (4.30–11.21)	<0.0001
		Iron protein succinylate	1	0	2.60 (1.20–4.00)	0.0003
		Compound iron preparations	3	18	5.53 (4.23–6.84)	<0.00001
MCH	Average age	<2.5 years	3	41	2.02 (1.18–2.85)	<0.00001
		≥2.5 years	2	0	2.04 (0.97–3.10)	0.0002
	Disease duration	<5 months	2	0	2.56 (1.68–3.44)	<0.00001
		≥5 months	2	0	1.90 (0.78–3.03)	0.0009
	Baseline Hb levels	<90 g/L	1	0	1.97 (0.51–3.43)	0.008
		≥90 g/L	3	56	3.06 (1.93–4.19)	<0.00001
	Treatment duration	4 weeks	3	41	2.07 (1.26–2.88)	<0.00001
		8 weeks	3	71	2.72 (1.13–4.32)	0.0008
	Type of iron supplementation	Iron dextran	3	41	2.07 (1.26–2.88)	<0.00001
		Iron protein succinylate	0	0	0	0
		Compound iron preparations	3	71	2.72 (1.13–4.32)	0.0008
SF	Average age	<2.5 years	2	0	5.58 (4.07–7.08)	<0.00001
		≥2.5 years	3	74	5.61 (3.09–8.13)	<0.0001
	Disease duration	<5 months	2	47	4.94 (2.71–7.16)	<0.0001
		≥5 months	3	62	6.04 (4.05–8.04)	<0.00001
	Baseline Hb levels	<90 g/L	2	0	5.43 (4.04–6.83)	<0.00001
		≥90 g/L	2	85	5.90 (1.55–10.25)	0.008
	Treatment duration	4 weeks	3	70	5.96 (3.61–8.31)	<0.00001
		8 weeks	2	0	5.02 (3.52–6.53)	<0.00001
	Type of iron supplementation	Iron dextran	2	85	5.90 (1.55–10.25)	0.008
		Iron protein succinylate	1	0	5.91 (3.94–7.88)	<0.00001
		Compound iron preparations	2	0	5.02 (3.52–6.53)	<0.00001
Total adverse events	Average age	<2.5 years	7	62	0.64 (0.28–1.49)	0.30
		≥2.5 years	3	0	0.24 (0.10–0.58)	0.001
	Disease duration	<5 months	5	58	0.65 (0.22–1.90)	0.43
		≥5 months	3	0	0.17 (0.07–0.45)	0.0004
	baseline Hb levels	<90 g/L	2	0	0.13 (0.04–0.41)	0.0006
		≥90 g/L	7	41	0.82 (0.43–1.55)	0.54
	Treatment duration	4 weeks	7	53	0.48 (0.22–1.02)	0.06
		8 weeks	4	53	0.59 (0.19–1.81)	0.36
	Probiotic strains	Single-strain	2	77	0.78 (0.20–2.96)	0.71
		Multi-strain	9	47	0.45 (0.22–0.92)	0.03
	Probiotic dosage	<1 × 10^7^ CFU/day	2	88	0.42 (0.03–5.94)	0.52
		≥1 × 10^7^ CFU/day	9	42	0.55 (0.29–1.01)	0.06
	Type of iron supplementation	Iron dextran	7	51	0.72 (0.36–1.42)	0.35
		Iron protein succinylate	1	0	0.11 (0.03–0.47)	0.003
		Compound iron preparations	3	17	0.37 (0.11–1.18)	0.09

MD, mean difference; CI, confidence interval; Hb, hemoglobin; RBC, red blood cell count; MCV, mean corpuscular volume; MCH, mean corpuscular hemoglobin; SF, serum ferritin.

Based on the weighted mean age reported in the included studies and to ensure balanced subgroup sizes, participants were divided into two age groups: < 2.5 years and ≥ 2.5 years. Subgroup analyses indicated that the heterogeneity of MCH was partly attributable to average age, whereas no corresponding reduction in heterogeneity was observed for the other outcomes. Specifically, probiotics substantially reduced MCH in both the < 2.5 years subgroup (MD 2.02 pg, 95% CI 1.18–2.85, *p* < 0.00001, *I*^2^ = 41%) and the ≥ 2.5 years subgroup (MD 2.04 pg, 95% CI 0.97–3.10, *p* = 0.0002, *I*^2^ = 0%).

For disease duration, participants were classified into two subgroups (< 5 months and ≥ 5 months) with reference to the weighted average duration reported in the included studies and with consideration for maintaining subgroup balance. Subgroup analyses indicated that the heterogeneity of MCH was related to disease duration, whereas no corresponding reduction in heterogeneity was observed for the other outcomes. Specifically, probiotics significantly reduced MCH in both the < 5 months subgroup (MD 2.56 pg, 95% CI 1.68–3.44, *p* < 0.00001, *I*^2^ = 0%) and the ≥ 5 months subgroup (MD 1.90 pg, 95% CI 0.78–3.03, *p* = 0.0009, *I*^2^ = 0%).

Studies were categorized into two subgroups based on baseline Hb levels: < 90 g/L and ≥ 90 g/L. Subgroup analyses indicated that the heterogeneity of total adverse events was related to baseline Hb levels, whereas no corresponding reduction in heterogeneity was observed for the other outcomes. Specifically, probiotics significantly reduced total adverse events in the < 90 g/L subgroup (RR 0.13, 95% CI 0.04–0.41, *p* = 0.0006, *I*^2^ = 0%), while no significant difference was observed in the ≥ 90 g/L subgroup (RR 0.82, 95% CI 0.43–1.55, *p* = 0.54, *I*^2^ = 41%).

Participants were divided into the 4 weeks and 8 weeks subgroups based on treatment duration. Subgroup analyses indicated that treatment duration did not account for the heterogeneity observed in Hb, RBC, MCV, MCH, SF, and total adverse events.

Participants were further categorized according to probiotic strains, forming single-strain and multi-strain subgroups. Subgroup analyses showed that differences in probiotic strains did not influence the heterogeneity of Hb, MCV, and total adverse events.

Stratification by probiotic dosage yielded two subgroups: < 1 × 10^7^ CFU/day and ≥ 1 × 10^7^ CFU/day. Subgroup analyses suggested that probiotic dosage had no discernible impact on the heterogeneity of Hb, RBC, MCV, and total adverse events.

Participants were stratified into three subgroups according to type of iron supplementation: iron dextran, iron protein succinylate, and compound iron preparations. Subgroup analyses revealed that the type of iron supplementation did not explain the heterogeneity observed in Hb, RBC, MCV, MCH, SF, and total adverse events.

In summary, the heterogeneity in MCH levels was partly attributable to differences in average age and disease duration, whereas the heterogeneity in total adverse events was partly related to baseline Hb levels. However, for Hb, RBC, MCV, and SF, the examined clinical factors did not fully explain the observed heterogeneity. Unmeasured study-level characteristics, including variations in probiotic preparation, storage conditions, participant management, and other insufficiently reported clinical factors, may also have contributed to the remaining heterogeneity. Because these variables were not consistently reported in the original studies, their effects could not be formally assessed. In addition, probiotic strain complexity was explored as a potential source of heterogeneity. However, for outcomes such as RBC, all included studies used multi-strain probiotic formulations; therefore, subgroup analysis based on strain type was not applicable for these outcomes.

### 3.6 Sensitivity analysis

The above analysis indicated that Hb, RBC, MCV, MCH, SF, and total adverse events demonstrated high heterogeneity (*I*^2^ ≥ 50%). Therefore, leave-one-out sensitivity analyses were conducted to investigate the sources of heterogeneity for these outcomes, as shown in Table 4.

**Table 4:** Leave-one-out sensitivity analyses of identified heterogeneous sources.

Outcome	Heterogeneity source	Sensitivity analysis results			Robustness
		*I*^2^/%	MD/RR (95% CI)	*p-*value	
MCH	Yang (40)	0	1.97 (1.45–2.49)	<0.00001	Robust
SF	Li (43)	0	5.05 (3.97–6.14)	<0.00001	Robust
Total adverse events	Han (36)	40	0.44 (0.24–0.81)	0.008	Robust

MD, mean difference; CI, confidence interval; MCH, mean corpuscular hemoglobin; SF, serum ferritin.

The sensitivity analysis revealed that the heterogeneity of MCH originated from the study by Yang et al., which may be attributed to the fact that the participants aged 1 to 7 years, in contrast to other studies that enrolled participants aged ≤ 5 years. After removing this study, the heterogeneity of MCH significantly decreased (*I*^2^ = 0), and the statistical significance remained unchanged (MD 1.97 pg, 95% CI 1.45–2.49, *p* < 0.00001), suggesting that the meta-analysis results for MCH were robust.

The sensitivity analysis indicated that the heterogeneity of SF was mainly driven by Li et al., likely because this study enrolled children aged 1.5 to 7 years with disease durations of 5 to 30 months, whereas the other studies included children aged ≤ 5 years with disease durations of ≤ 9 months. After excluding this study, heterogeneity decreased substantially (*I*^2^ = 0), and the statistical significance remained unchanged (MD 5.05 μg/L, 95% CI 3.97–6.14, *p* < 0.00001), suggesting that the meta-analysis results for SF were robust.

The sensitivity analysis showed that the heterogeneity of total adverse events originated from the study by Han et al. This may be explained by statistical heterogeneity, as no notable clinical or methodological differences were observed compared with the other studies. After excluding this study, heterogeneity decreased substantially (*I*^2^ = 40%), and the statistical significance remained unchanged (RR 0.44, 95% CI 0.24–0.81, *p* = 0.008), suggesting that the meta-analysis results for total adverse events were robust.

In summary, the heterogeneity in MCH and SF levels was partly attributable to differences in participants’ average age and disease duration. However, for Hb, RBC, MCV, and total adverse events, these clinical factors could not fully explain the observed heterogeneity, which was likely driven by statistical heterogeneity across the included studies.

### 3.7 TSA

**Figure 9:**
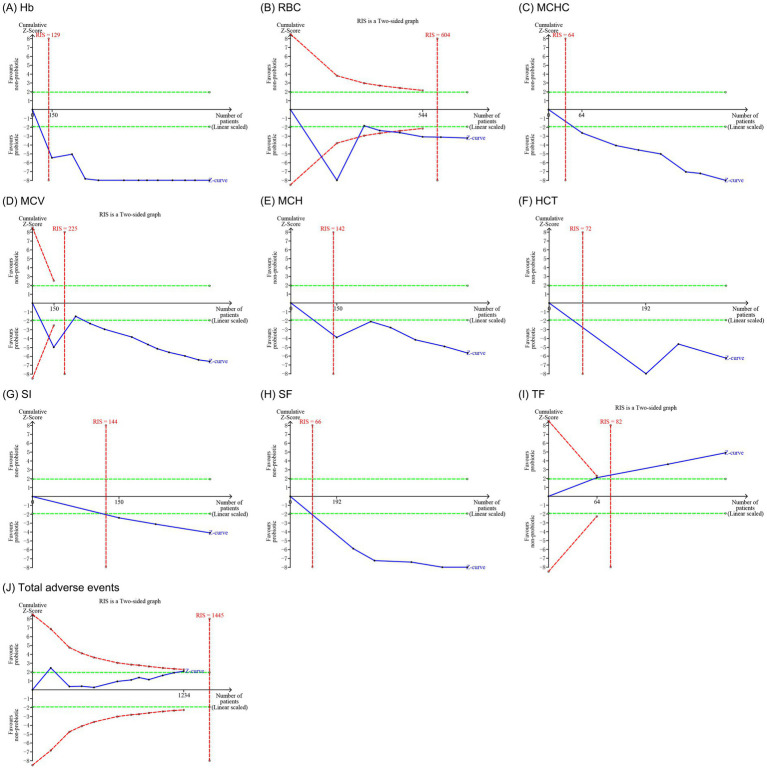
Trial sequential analyses of statistically significant outcomes: **(A)** Hb; **(B)** RBC; **(C)** MCHC; **(D)** MCV; **(E)** MCH; **(F)** HCT; **(G)** SI; **(H)** SF; **(I)** TF; **(J)** total adverse events. Hb, hemoglobin; RBC, red blood cell count; MCHC, mean corpuscular hemoglobin concentration; MCV, mean corpuscular volume; MCH, mean corpuscular hemoglobin; HCT, hematocrit; SI, serum iron; SF, serum ferritin; TF, transferrin.

The TSA was conducted to assess the robustness of statistically significant findings and to reduce the risk of false-positive results caused by sparse data and repeated significance testing. The cumulative Z-curves for Hb, RBC, MCHC, MCV, MCH, HCT, SI, SF, and TF crossed the predefined monitoring boundaries, indicating that the evidence for these outcomes has reached conclusiveness. In contrast, the cumulative Z-curve for total adverse events did not cross the monitoring boundaries. The RIS was estimated at 1,445 participants, while the cumulative sample size was 1,234, indicating that the current evidence remains underpowered and should be interpreted with caution. The TSA results are shown in Figure 9.

### 3.8 Publication bias

**Figure 10:**
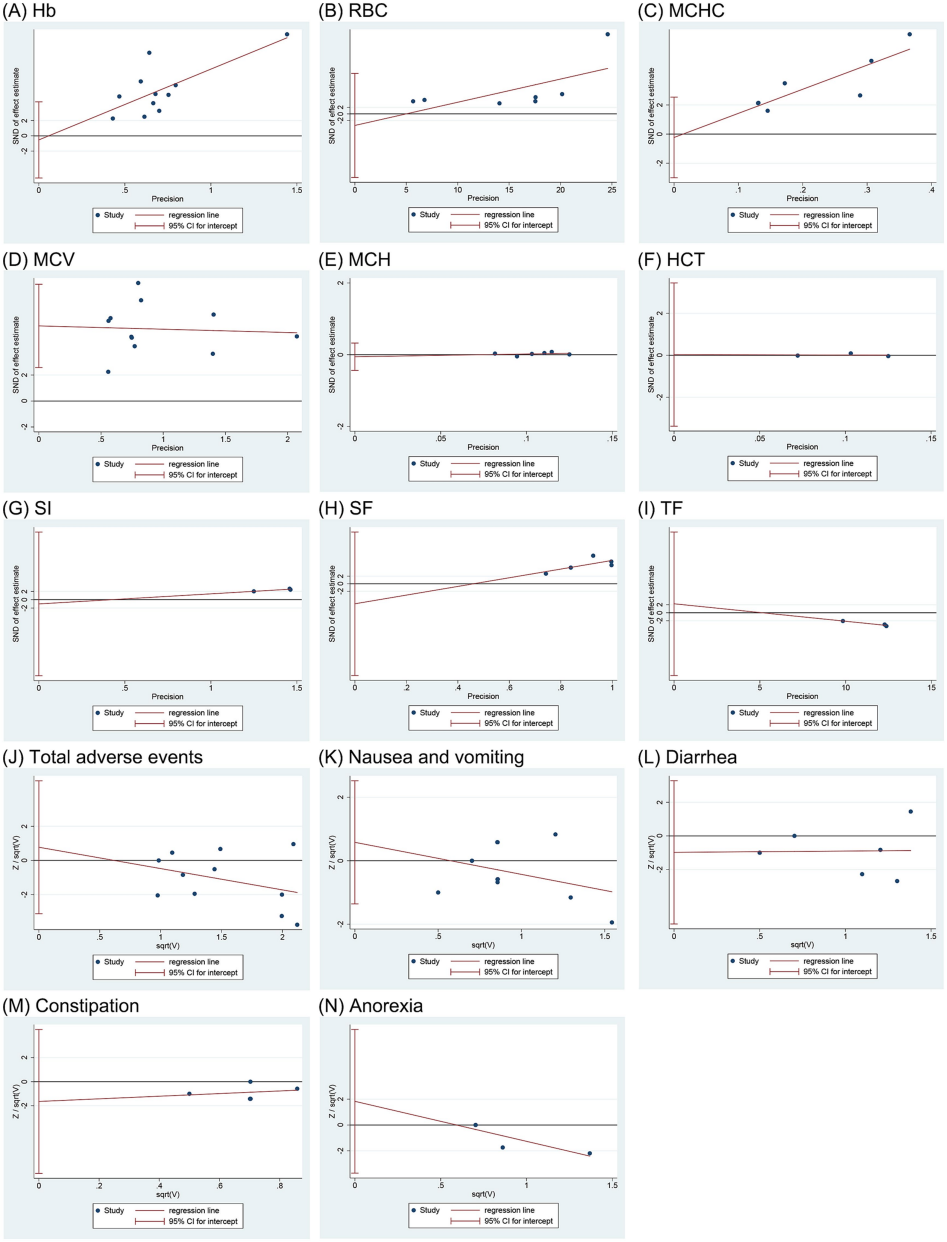
Egger’s and Harbord’s tests of publication bias: **(A)** Hb; **(B)** RBC; **(C)** MCHC; **(D)** MCV; **(E)** MCH; **(F)** HCT; **(G)** SI; **(H)** SF; **(I)** TF; **(J)** total adverse events; **(K)** nausea and vomiting; **(L)** diarrhea; **(M)** constipation; **(N)** anorexia. Hb, hemoglobin; RBC, red blood cell count; MCHC, mean corpuscular hemoglobin concentration; MCV, mean corpuscular volume; MCH, mean corpuscular hemoglobin; HCT, hematocrit; SI, serum iron; SF, serum ferritin; TF, transferrin.

Publication bias was assessed using Egger’s test for Hb, RBC, MCHC, MCV, MCH, HCT, SI, SF, and TF levels, and Harbord’s test for total adverse events, nausea and vomiting, diarrhea, constipation, and anorexia, as depicted in Figure 10. No significant publication bias was detected for Hb (*p* = 0.815), RBC (*p* = 0.593), MCHC (*p* = 0.839), MCH (*p* = 0.702), HCT (*p* = 0.918), SI (*p* = 0.593), SF (*p* = 0.439), TF (*p* = 0.357), total adverse events (*p* = 0.664), nausea and vomiting (*p* = 0.516), diarrhea (*p* = 0.581), constipation (*p* = 0.447), and anorexia (*p* = 0.292). However, significant publication bias was observed for MCV (*p* = 0.003). Trim-and-fill analysis imputed one potentially missing study, and the adjusted pooled effect remained consistent with the original findings, suggesting that publication bias had little influence on the overall conclusion. The Trim-and-fill results are shown in Supplementary Figure S1. Because only two studies reported abdominal pain and metallic taste, publication bias was not assessed for these outcomes. Nevertheless, publication bias tests such as Egger’s and Harbord’s tests generally have limited statistical power when fewer than 10 studies are included. Therefore, the results for outcomes based on a small number of studies should be interpreted with caution.

### 3.9 Certainty of evidence

According to the GRADE assessment, the certainty of evidence was rated as low for MCHC, HCT, SI, and TF, whereas it was rated as very low for Hb, RBC, MCV, MCH, SF, total adverse events, anorexia, nausea and vomiting, metallic taste, abdominal pain, diarrhea, and constipation. Overall, the certainty of evidence ranged from low to very low across all outcomes, indicating limited confidence in the estimated effects. Detailed GRADE ratings are summarized in Table 5.

**Table 5:** GRADE summary of findings for the included outcomes.

Outcome	Studies (participants)	Risk of bias	Inconsistency	Indirectness	Imprecision	Publication bias	MD/RR (95% CI)	Certainty of evidence
Hb	12 (1,342)	Very serious	Serious	None	None	None	8.64 (6.77–10.52)	Very low
RBC	7 (728)	Very serious	Serious	None	None	None	0.47 (0.19–0.76)	Very low
MCHC	7 (790)	Very serious	None	None	None	None	15.66 (12.55–18.77)	Low
MCV	11 (1,234)	Very serious	Serious	None	None	Serious	6.68 (4.69–8.67)	Very low
MCH	6 (578)	Very serious	Serious	None	None	None	2.40 (1.57–3.22)	Very low
HCT	3 (350)	Very serious	None	None	None	None	3.36 (2.31–4.41)	Low
SI	3 (309)	Very serious	None	None	None	None	1.70 (0.89–2.51)	Low
SF	5 (538)	Very serious	Serious	None	None	None	5.62 (4.24–7.00)	Very low
TF	3 (234)	Very serious	None	None	None	None	–0.24 (–0.34 to –0.15)	Low
Total adverse events	11 (1,234)	Very serious	Serious	None	Serious	None	0.52 (0.28–0.97)	Very low
Anorexia	4 (504)	Very serious	None	None	Serious	None	0.33 (0.09–1.18)	Very low
Nausea and vomiting	11 (1,234)	Very serious	None	None	Serious	None	0.68 (0.36–1.28)	Very low
Metallic taste	2 (302)	Very serious	None	None	Serious	Suspected	0.39 (0.10–1.45)	Very low
Abdominal pain	2 (206)	Very serious	None	None	Serious	Suspected	0.40 (0.06–2.70)	Very low
Diarrhea	7 (846)	Very serious	None	None	Serious	None	0.51 (0.17–1.53)	Very low
Constipation	5 (428)	Very serious	None	None	Serious	None	0.39 (0.11–1.38)	Very low

MD, mean difference; CI, confidence interval; Hb, hemoglobin; RBC, red blood cell count; MCHC, mean corpuscular hemoglobin concentration; MCV, mean corpuscular volume; MCH, mean corpuscular hemoglobin; HCT, hematocrit; SI, serum iron; SF, serum ferritin; TF, transferrin.

## 4 Discussion

### 4.1 Background and principal findings

IDA is one of the most common types of anemia worldwide, particularly affecting children and pregnant women (47). Two previous meta-analyses have suggested that probiotics may improve anemia-related outcomes and promote iron absorption (19, 21). However, the meta-analysis by Vonderheid et al. (19) did not distinguish between adult and pediatric populations, which may have introduced additional confounding and compromised the consistency and credibility of the findings. In contrast, the meta-analysis conducted by Tremblay et al. (21) included only three clinical trials, and the limited sample size reduced the precision and external validity of the results.

To address these limitations, the present study comprehensively searched both international and Chinese databases, expanded the range of outcome measures, and aimed to provide a more precise evaluation of the efficacy of probiotics in Chinese children with IDA. Our results showed that probiotics significantly increased Hb, RBC, MCHC, MCV, MCH, HCT, SI, and SF levels, while reducing TF levels and total adverse events. TSA demonstrated that, except for total adverse events, all outcomes showing significant between-group differences reached conclusiveness, thereby strengthening the reliability of the evidence. According to the GRADE assessment, the certainty of evidence for MCHC, HCT, SI, and TF was rated as low, while the certainty for the remaining outcomes was very low, indicating that these findings should be interpreted cautiously.

### 4.2 Efficacy and safety assessment

IDA is a typical microcytic hypochromic anemia characterized by reduced Hb, MCH, and MCV (48). This analysis showed that probiotics significantly increased Hb by 8.64 g/L, RBC by 0.47 × 10^12^/L, MCHC by 15.66 g/L, MCV by 6.68 fL, MCH by 2.40 pg, and HCT by 3.36%. Among these outcomes, improvements in Hb, RBC, and HCT reflect effective alleviation of anemia, while increases in MCV, MCH, and MCHC indicate a shift of erythrocytes from a microcytic, hypochromic pattern toward a more normocytic and normochromic profile. Consistent with our findings, Tremblay et al. (21) reported that probiotics significantly improved the treatment response rate of IDA (RR 0.51, 0.28–0.92). Subgroup analyses suggested that heterogeneity in MCH may be attributable to differences in average age and disease duration among the included populations. On the one hand, infants are more susceptible to IDA under iron-deficient conditions than older children (49, 50). Specifically, infants exhibit active erythropoiesis with limited iron reserves, making them more prone to developing microcytic hypochromic anemia (49, 50). In contrast, older children generally have relatively sufficient iron stores, and even in the presence of iron deficiency, the decline in MCH may be less pronounced (49, 50). On the other hand, prolonged iron deficiency affects the size and hemoglobin content of newly produced erythrocytes. During short-term iron deficiency, erythrocytes with normal size and hemoglobin content may still circulate, resulting in less obvious reductions in MCH, whereas long-term deficiency predominantly leads to the production of microcytic hypochromic erythrocytes with markedly reduced MCH (51). Sensitivity analysis indicated that heterogeneity in MCH was mainly driven by the study conducted by Yang et al. (40), which may be explained by differences in average age. After excluding this study, the pooled effect for MCH remained statistically significant (MD 1.97 pg, 95% CI 1.45–2.49, *p* < 0.00001), indicating that the result was robust. In addition, differences in probiotic strain composition were explored as a potential source of variability. For hemoglobin, subgroup analysis comparing single-strain and multi-strain formulations indicated that stratification by strain composition did not substantially reduce heterogeneity, suggesting that strain complexity is unlikely to be a major determinant of the observed variability. For red blood cell count, all included studies used multi-strain formulations; therefore, the effect of strain composition could not be further assessed. Overall, the remaining heterogeneity may reflect the influence of unmeasured clinical and methodological factors rather than strain-related differences alone.

Notably, among all evaluated outcomes, potential publication bias was observed only for MCV. First, MCV is highly susceptible to baseline heterogeneity across study populations. Variations in baseline MCV values, as well as differences in the severity and duration of iron deficiency and nutritional status, may substantially influence the magnitude of treatment response. In particular, studies enrolling patients with markedly reduced baseline MCV levels are more likely to report larger effect sizes, thereby increasing inter-study variability. Second, the treatment duration across the included studies ranged from 4 to 8 weeks, which may have been insufficient to fully capture the recovery kinetics of MCV. Compared with other hematological and iron metabolism parameters, MCV responds relatively slowly to iron supplementation because preexisting microcytic erythrocytes remain in the circulation throughout their normal lifespan following treatment (52). Consequently, normalization of MCV generally lags behind improvements in hemoglobin and iron metabolism indices. Third, the relatively small sample sizes of the included studies may have further increased the likelihood of unstable effect estimates. Collectively, these factors may have contributed to the potential presence of publication bias for MCV. Nevertheless, sensitivity analyses demonstrated that the statistical significance of MCV remained stable after sequential exclusion of individual studies, supporting the robustness of the pooled findings. Furthermore, TSA confirmed that the cumulative evidence for MCV had reached conclusiveness. Taken together, although the overall findings appear robust, the pooled effect estimate for MCV should still be interpreted with caution given the potential presence of publication bias, and further large-scale, rigorously designed studies are warranted to validate these findings.

Iron deficiency represents another hallmark of IDA and is characterized by decreased SI and SF levels and increased TF levels (53, 54). SI reflects the amount of circulating iron available for erythropoiesis, and low levels indicate insufficient iron supply (55, 56). SF reflects body iron stores, and its reduction indicates depletion of iron reserves, making it one of the most sensitive indicators of iron deficiency (57, 58). TF is the major iron transport protein responsible for delivering iron to the bone marrow for erythropoiesis and is typically upregulated as a compensatory response to iron deficiency (54). This analysis showed that probiotics increased SI by 1.70 μmol/L and SF by 5.62 μg/L, while reducing TF by 0.24 g/L, suggesting an overall improvement in iron metabolism. In a previous meta-analysis, Vonderheid et al. (19) reported that *Lactobacillus plantarum* 299v promoted iron absorption (SMD 0.55, 95% CI 0.22–0.88), which is consistent with our findings. Unlike prior studies that focused primarily on iron absorption, our analysis evaluated a broader range of iron metabolism outcomes, including SI, SF, and TF. Sensitivity analysis indicated that heterogeneity in SF was mainly driven by the study conducted by Li et al. (43), which included participants with a wider age range (1.5–7 years) and longer disease duration (5–30 months), whereas the other studies restricted enrollment to children aged ≤ 5 years with disease duration ≤ 9 months. Exclusion of this study did not materially change the pooled estimate (MD 5.05 μg/L, 95% CI 3.97–6.14, *p* < 0.00001), indicating that the result was robust. Collectively, these findings make it evident that probiotics can enhance iron metabolism in children with IDA.

Furthermore, our analysis showed that probiotic supplementation was associated with a 48% reduction in total adverse events. This finding is consistent with a previous meta-analysis reporting that probiotics combined with iron supplementation reduced adverse events by 47% compared with iron supplementation alone (RR 0.53, 0.37–0.77) (21). Subgroup analyses indicated that the heterogeneity observed in total adverse events may be associated with variations in baseline Hb levels among the included participants. This finding may be explained by the fact that children with lower baseline Hb levels generally had more severe iron deficiency and gastrointestinal dysfunction, thereby potentially deriving greater gastrointestinal benefits from probiotic supplementation during iron therapy. Sensitivity analysis identified the study by Han et al. (36) as the primary source of heterogeneity for total adverse events. However, exclusion of this study did not materially alter the pooled estimate (RR 0.44, 95% CI 0.24–0.81, *p* = 0.008), indicating that the result was relatively stable. As no apparent clinical or methodological differences were observed between this study and the others, the heterogeneity was considered to be mainly statistical in nature. Notably, although a significant reduction was observed for total adverse events, no statistically significant differences were detected for individual adverse events, including anorexia, nausea and vomiting, metallic taste, abdominal pain, diarrhea, and constipation. Importantly, TSA conducted specifically for total adverse events showed that the cumulative Z-curve did not cross the monitoring boundary or reach the RIS, indicating that the current evidence remains insufficient to draw firm conclusions regarding safety. This inconsistency between conventional meta-analysis and TSA findings suggests that the observed reduction in total adverse events may be influenced by limited sample size and random error. In addition, The absence of allocation concealment and blinding in the included trials may have influenced the evaluation of safety outcomes, particularly those relying on subjective symptom reporting such as gastrointestinal tolerability. Awareness of treatment allocation could have shaped participants’ perception of symptoms and their willingness to report mild gastrointestinal events, while also affecting investigators’ interpretation of non-specific complaints. Such context-dependent reporting may introduce variability in adverse event ascertainment, thereby affecting the robustness of the observed safety profile and its interpretation. Therefore, the potential safety benefits of probiotics should be interpreted with caution and require confirmation in large-scale, multicenter RCTs.

### 4.3 Mechanistic considerations

Probiotics may regulate iron metabolism through multiple complementary mechanisms. First, probiotics such as *Lactobacillus* and *Bifidobacterium* strains produce metabolic byproducts including lactic acid, acetic acid, and butyric acid, which lower intestinal pH and increase short-chain fatty acid levels, thereby facilitating the reduction of non-heme iron from ferric iron to the more readily absorbable ferrous iron (19, 59). Second, probiotics may enhance intestinal iron uptake by upregulating epithelial iron transporters, such as divalent metal transporter 1 (DMT1) (59), thereby facilitating dietary iron uptake into enterocytes. In contrast, cellular uptake of circulating iron is predominantly mediated by transferrin receptor 1 (TFRC), which enables the internalization of transferrin-bound iron from the circulation and its subsequent release within endosomes for intracellular utilization (60). Together, DMT1-mediated intestinal absorption and TFRC-mediated cellular iron uptake represent complementary processes that coordinate iron entry and utilization, highlighting the coordinated role of gastrointestinal absorption and peripheral cellular iron acquisition in maintaining systemic iron homeostasis. Third, probiotics have been shown to suppress hepcidin expression, a key regulator that restricts iron absorption; downregulation of hepcidin may subsequently promote the activity of ferroportin 1, allowing greater iron export into the circulation (61, 62). Fourth, probiotics may strengthen intestinal barrier integrity by increasing the expression of mucins and tight junction proteins, thereby improving barrier function and attenuating Lipopolysaccharide induced inflammatory responses, which provides a more stable microenvironment for iron absorption (63, 64). Fifth, probiotics may inhibit competition for iron by siderophore dependent pathogenic bacteria, reducing iron sequestration by pathogens at the ecological niche level and further enhancing iron bioavailability (65). Collectively, these mechanisms suggest that probiotics promote iron absorption and utilization through coordinated modulation of the intestinal microenvironment, iron transport pathways, inflammatory responses, and microbial competition.

### 4.4 Clinical implications

This meta-analysis provides several clinically relevant implications for the management of pediatric IDA. First, the concordant improvements observed in Hb, RBC, MCHC, MCV, MCH, and HCT suggest a global enhancement of erythropoiesis, supporting the potential role of microbiota-targeted interventions in anemia management. In parallel, the favorable effects on SI, SF, and TF extend current evidence beyond iron absorption alone and imply a broader regulatory effect of probiotics on iron metabolism and iron homeostasis. Notably, Hb, the primary indicator of anemia treatment response, showed a pooled mean increase of 8.64 g/L. For clinical interpretation, a hemoglobin increase of 10 g/L (1.0 g/dL), commonly regarded as the minimal clinically important difference, was used as the reference threshold (66). Although the improvement was statistically significant, it did not reach this threshold, suggesting that the clinical benefit may be modest. Nevertheless, probiotics were administered as an adjunct to conventional iron therapy rather than as a standalone treatment, and consistent improvements were also observed across multiple hematologic and iron metabolism parameters. Therefore, despite not reaching the reference threshold for a clinically meaningful Hb response, these findings support a potential complementary role for probiotics in promoting hematologic recovery and improving iron metabolism in children with IDA.

In terms of safety, probiotics were associated with a reduction in total adverse events; however, the absence of significant effects on individual gastrointestinal symptoms and the inconclusive TSA findings indicate that the safety advantage should be interpreted cautiously. Overall, probiotics may represent a beneficial complementary strategy for pediatric IDA management, although further large-scale and high-quality trials are required to determine whether these hematologic improvements translate into clinically important long-term outcomes.

### 4.5 Limitations and prospects

Several limitations of this study should be acknowledged. First, the number of included RCTs was limited, and the overall sample size was relatively small. In particular, the cumulative sample size for total adverse events did not reach the RIS, which may have compromised the precision of the estimates. Second, none of the included studies clearly reported allocation concealment or blinding, which may introduce performance and detection bias, particularly for subjective outcomes such as gastrointestinal tolerability, thereby affecting safety assessment. Third, all included studies were conducted exclusively in Chinese children, which may limit the generalizability of the findings. This is because differences in dietary patterns may influence iron intake and absorption, while variations in gut microbiota composition may affect iron metabolism and probiotic activity. In addition, genetic and environmental factors, as well as differences in healthcare delivery systems, may further contribute to heterogeneity in treatment response. Therefore, caution is warranted when extrapolating these findings to other populations. Fourth, all available data were derived from short-term interventions, precluding assessment of the long-term efficacy of probiotic supplementation in children with IDA. Fifth, although publication bias was assessed using Egger’s test and Harbord’s test, the number of included studies for some outcomes was relatively small. Sixth, None of the included randomized controlled trials had preregistered protocols available. This methodological limitation reduces transparency and increases the potential for selective outcome reporting bias, including the possible underrepresentation of non-significant outcomes. Therefore, the statistical power and reliability of these tests may have been limited, and the results should be interpreted with caution. Future research should prioritize large-scale, well-designed, multicenter RCTs with longer follow-up periods, with particular attention to safety and sustained efficacy. In addition, detailed stratified analyses based on participant characteristics and intervention features are warranted to support the development of more generalizable and individualized treatment strategies.

## 5 Conclusion

Probiotics appear to improve anemia-related parameters and iron metabolism in Chinese children with IDA. However, the improvement in Hb levels did not exceed the minimal clinically important difference, and the TSA results for total adverse events remained inconclusive. Therefore, probiotics should be considered as an adjunctive therapy to oral iron supplementation rather than a standalone intervention. Furthermore, given the potential selection and performance biases, as well as the exclusive inclusion of Chinese children, these findings should be interpreted with caution. Further rigorously designed, adequately powered multicenter studies involving ethnically and geographically diverse populations are needed to validate these findings.

## Data Availability

The original contributions presented in the study are included in the article/Supplementary material, further inquiries can be directed to the corresponding authors.

## Glossary

IDA: Iron deficiency anemia
TSA: Trial sequential analysis
Hb: Hemoglobin
RBC: Red blood cell
MCHC: Mean corpuscular hemoglobin concentration
MCV: Mean corpuscular volume
MCH: Mean corpuscular hemoglobin
HCT: Hematocrit
SI: Serum iron
SF: Serum ferritin
TF: Transferrin
CNKI: China National Knowledge Infrastructure
CSTJ: China Science and Technology Journal Database
ChiCTR: Chinese Clinical Trial Registry
WHO ICTRP: World Health Organization International Clinical Trials Registry Platform
MD: Mean difference
RR: Risk ratio
PRISMA: Preferred Reporting Items for Systematic Reviews and Meta-Analyses
RCTs: Randomized controlled trials
RIS: Required information size
MeSH: Medical Subject Headings
CI: Confidence interval
GRADE: Grading of Recommendations, Assessment, Development, and Evaluation
DMT1: Divalent metal transporter 1
TFRC: Transferrin receptor 1
