# Supplementary material for: Efficacy and safety of probiotics in the treatment of iron deficiency anemia in Chinese children: a systematic review and meta-analysis
Source: Front Nutr. 2026 Jul 30;13:1876092. doi: 10.3389/fnut.2026.1876092 (PMC13468811; doi:10.3389/fnut.2026.1876092)
Supplement: Supplementary file 1 [file Table_1.DOCX]

**Supplementary Table S1** The search strategies, filters, and limits for each database.

| Database | Search strategy | Run dates | Filters | Limits | Records |
| --- | --- | --- | --- | --- | --- |
| PubMed | (Iron Deficiency Anemia[Title/Abstract] OR Iron Deficiency Anemias[Title/Abstract] OR Iron-Deficiency Anemia[Title/Abstract] OR Iron Deficiency Anemia[Title/Abstract]) AND (Probiotics[Title/Abstract] OR Probiotic[Title/Abstract] OR Bifidobacterium[Title/Abstract] OR Bifidobacteria[Title/Abstract] OR Bacillus bifida[Title/Abstract] OR Yeast[Title/Abstract] OR Saccharomyces cerevisiae[Title/Abstract] OR Saccharomyces italicus[Title/Abstract] OR Saccharomyces oviformis[Title/Abstract] OR S cerevisiae[Title/Abstract] OR S. cerevisiae[Title/Abstract] OR Saccharomyces uvarum var melibiosus[Title/Abstract] OR Candida robusta[Title/Abstract] OR Saccharomyces capensis[Title/Abstract] OR Lactobacillus acidophilus[Title/Abstract] OR Lactobacillus amylovorus[Title/Abstract] OR Lactobacill[Title/Abstract] OR Lactic acid bacteria[Title/Abstract] OR Clostridium butyricum[Title/Abstract] OR Bacillus[Title/Abstract] OR Natto Bacteria[Title/Abstract] OR Streptococcus thermophiles[Title/Abstract] OR Enterococcus[Title/Abstract]) | April 30, 2026 | None | None | 56 |
| Embase | ('iron deficiency anemias':ab,ti OR 'iron-deficiency anemia':ab,ti OR 'iron deficiency anemia':ab,ti) AND (probiotics:ab,ti OR probiotic:ab,ti OR bifidobacterium:ab,ti OR bifidobacteria:ab,ti OR 'bacillus bifida':ab,ti OR yeast:ab,ti OR 'saccharomyces cerevisiae':ab,ti OR 'saccharomyces italicus':ab,ti OR 'saccharomyces oviformis':ab,ti OR 's cerevisiae':ab,ti OR 's. cerevisiae':ab,ti OR 'saccharomyces uvarum var melibiosus':ab,ti OR 'candida robusta':ab,ti OR 'saccharomyces capensis':ab,ti OR 'lactobacillus acidophilus':ab,ti OR 'lactobacillus amylovorus':ab,ti OR lactobacill:ab,ti OR 'lactic acid bacteria':ab,ti OR 'clostridium butyricum':ab,ti OR bacillus:ab,ti OR 'natto bacteria':ab,ti OR 'streptococcus thermophiles':ab,ti OR enterococcus:ab,ti) | April 30, 2026 | None | None | 97 |
| Cochrane Library | Iron Deficiency Anemia OR Iron Deficiency Anemias OR Iron-Deficiency Anemia OR Iron Deficiency Anemia in Title Abstract Keyword AND Probiotics OR Probiotic OR Bifidobacterium OR Bifidobacteria OR Bacillus bifida OR Yeast OR Saccharomyces cerevisiae OR Saccharomyces italicus OR Saccharomyces oviformis OR S cerevisiae OR S. cerevisiae OR Saccharomyces uvarum var melibiosus OR Candida robusta OR Saccharomyces capensis OR Lactobacillus acidophilus OR Lactobacillus amylovorus OR Lactobacill OR Lactic acid bacteria OR Clostridium butyricum OR Bacillus OR Natto Bacteria OR Streptococcus thermophiles OR Enterococcus in Title Abstract Keyword | April 30, 2026 | None | None | 68 |
| Web of Science | Iron Deficiency Anemia OR Iron Deficiency Anemias OR Iron-Deficiency Anemia OR Iron Deficiency Anemia (Topic) AND Probiotics OR Probiotic OR Bifidobacterium OR Bifidobacteria OR Bacillus bifida OR Yeast OR Saccharomyces cerevisiae OR Saccharomyces italicus OR Saccharomyces oviformis OR S cerevisiae OR S. cerevisiae OR Saccharomyces uvarum var melibiosus OR Candida robusta OR Saccharomyces capensis OR Lactobacillus acidophilus OR Lactobacillus amylovorus OR Lactobacill OR Lactic acid bacteria OR Clostridium butyricum OR Bacillus OR Natto Bacteria OR Streptococcus thermophiles OR Enterococcus (Topic) | April 30, 2026 | None | None | 199 |
| ClinicalTrials.gov | (Iron Deficiency Anemia OR Iron Deficiency Anemias OR Iron-Deficiency Anemia OR Iron Deficiency Anemia) AND (Probiotics OR Probiotic OR Bifidobacterium OR Bifidobacteria OR Bacillus bifida OR Yeast OR Saccharomyces cerevisiae OR Saccharomyces italicus OR Saccharomyces oviformis OR S cerevisiae OR S. cerevisiae OR Saccharomyces uvarum var melibiosus OR Candida robusta OR Saccharomyces capensis OR Lactobacillus acidophilus OR Lactobacillus amylovorus OR Lactobacill OR Lactic acid bacteria OR Clostridium butyricum OR Bacillus OR Natto Bacteria OR Streptococcus thermophiles OR Enterococcus) | April 30, 2026 | None | None | 12 |
| WHO ICTRP | (Iron Deficiency Anemia OR Iron Deficiency Anemias OR Iron-Deficiency Anemia OR Iron Deficiency Anemia) AND (Probiotics OR Probiotic OR Bifidobacterium OR Bifidobacteria OR Bacillus bifida OR Yeast OR Saccharomyces cerevisiae OR Saccharomyces italicus OR Saccharomyces oviformis OR S cerevisiae OR S. cerevisiae OR Saccharomyces uvarum var melibiosus OR Candida robusta OR Saccharomyces capensis OR Lactobacillus acidophilus OR Lactobacillus amylovorus OR Lactobacill OR Lactic acid bacteria OR Clostridium butyricum OR Bacillus OR Natto Bacteria OR Streptococcus thermophiles OR Enterococcus) | April 30, 2026 | None | None | 1 |
| CNKI | TKA=((缺铁性贫血+铁缺乏性贫血+营养性贫血)*(益生菌+微生态制剂+双歧杆菌+酵母菌+嗜酸乳杆菌+乳酸菌+酪酸梭菌+丁酸梭菌+芽孢杆菌+枯草杆菌+链球菌)) | April 30, 2026 | None | None | 51 |
| CSTJ | M=((缺铁性贫血+铁缺乏性贫血+营养性贫血)*(益生菌+微生态制剂+双歧杆菌+酵母菌+嗜酸乳杆菌+乳酸菌+酪酸梭菌+丁酸梭菌+芽孢杆菌+枯草杆菌+链球菌)) | April 30, 2026 | None | None | 39 |
| Wanfang | 主题: ((缺铁性贫血 OR 铁缺乏性贫血 OR 营养性贫血) AND (益生菌 OR 微生态制剂 OR 双歧杆菌 OR 酵母菌 OR 嗜酸乳杆菌 OR 乳酸菌 OR 酪酸梭菌 OR 丁酸梭菌 OR 芽孢杆菌 OR 枯草杆菌 OR 链球菌)) | April 30, 2026 | None | None | 81 |
| ChiCTR | 研究疾病名称: 缺铁性贫血 | April 30, 2026 | None | None | 38 |

CNKI, China National Knowledge Infrastructure ; CSTJ, China Science and Technology Journal Database; Chinese Clinical Trial Registry (ChiCTR); World Health Organization International Clinical Trials Registry Platform (WHO ICTRP).
